# A reduced-carbohydrate and lactose-free formulation for stabilization among hospitalized children with severe acute malnutrition: A double-blind, randomized controlled trial

**DOI:** 10.1371/journal.pmed.1002747

**Published:** 2019-02-26

**Authors:** Robert H. J. Bandsma, Wieger Voskuijl, Emmanuel Chimwezi, Greg Fegan, André Briend, Johnstone Thitiri, Moses Ngari, Laura Mwalekwa, Victor Bandika, Rehema Ali, Fauzat Hamid, Betty Owor, Neema Mturi, Isabel Potani, Benjamin Allubha, Anneke C. Muller Kobold, Rosalie H. Bartels, Christian J. Versloot, Marjon Feenstra, Deborah A. van den Brink, Patrick F. van Rheenen, Marko Kerac, Celine Bourdon, James A. Berkley

**Affiliations:** 1 Division of Gastroenterology, Hepatology and Nutrition, Hospital for Sick Children, Toronto, Canada; 2 Translational Medicine Program, Hospital for Sick Children, Toronto, Canada; 3 Center for Global Child Health, Hospital for Sick Children, Toronto, Canada; 4 Department of Nutrition Sciences, University of Toronto, Toronto, Canada; 5 University of Groningen, University Medical Center Groningen, Department of Pediatrics, Groningen, the Netherlands; 6 Department of Biomedical Sciences, College of Medicine, University of Malawi, Blantyre, Malawi; 7 The Childhood Acute Illness and Nutrition Network (CHAIN), Nairobi, Kenya; 8 Department of Paediatrics and Child Health College of Medicine, University of Malawi, Blantyre, Malawi; 9 Global Child Health Group, Emma Children’s Hospital, Academic Medical Centre, Amsterdam, the Netherlands; 10 Swansea Trials Unit, Swansea University Medical School, Swansea, United Kingdom; 11 Department of Nutrition, Exercise and Sports, University of Copenhagen, Denmark; 12 University of Tampere School of Medicine, Center for Child Health Research, Tampere, Finland; 13 KEMRI/Wellcome Trust Research Programme, Kilifi, Kenya; 14 Department of Paediatrics, Coast General Hospital, Mombasa, Kenya; 15 University of Groningen, University Medical Center Groningen, Department of Laboratory Medicine, Groningen, the Netherlands; 16 London School of Hygiene & Tropical Medicine, London, United Kingdom; 17 Centre for Tropical Medicine and Global Health, Nuffield Department of Clinical Medicine, University of Oxford, United Kingdom; London School of Hygiene and Tropical Medicine, UNITED KINGDOM

## Abstract

**Background:**

Children with medically complicated severe acute malnutrition (SAM) have high risk of inpatient mortality. Diarrhea, carbohydrate malabsorption, and refeeding syndrome may contribute to early mortality and delayed recovery. We tested the hypothesis that a lactose-free, low-carbohydrate F75 milk would serve to limit these risks, thereby reducing the number of days in the stabilization phase.

**Methods and findings:**

In a multicenter double-blind trial, hospitalized severely malnourished children were randomized to receive standard formula (F75) or isocaloric modified F75 (mF75) without lactose and with reduced carbohydrate. The primary endpoint was time to stabilization, as defined by the World Health Organization (WHO), with intention-to-treat analysis. Secondary outcomes included in-hospital mortality, diarrhea, and biochemical features of malabsorption and refeeding syndrome. The trial was registered at clinicaltrials.gov (NCT02246296). Four hundred eighteen and 425 severely malnourished children were randomized to F75 and mF75, respectively, with 516 (61%) enrolled in Kenya and 327 (39%) in Malawi. Children with a median age of 16 months were enrolled between 4 December 2014 and 24 December 2015. One hundred ninety-four (46%) children assigned to F75 and 188 (44%) to mF75 had diarrhea at admission. Median time to stabilization was 3 days (IQR 2–5 days), which was similar between randomized groups (0.23 [95% CI −0.13 to 0.60], *P* = 0.59). There was no evidence of effect modification by diarrhea at admission, age, edema, or HIV status. Thirty-six and 39 children died before stabilization in the F75 and in mF75 arm, respectively (*P* = 0.84). Cumulative days with diarrhea (*P* = 0.27), enteral (*P* = 0.42) or intravenous fluids (*P* = 0.19), other serious adverse events before stabilization, and serum and stool biochemistry at day 3 did not differ between groups. The main limitation was that the primary outcome of clinical stabilization was based on WHO guidelines, comprising clinical evidence of recovery from acute illness as well as metabolic stabilization evidenced by recovery of appetite.

**Conclusions:**

Empirically treating hospitalized severely malnourished children during the stabilization phase with lactose-free, reduced-carbohydrate milk formula did not improve clinical outcomes. The biochemical analyses suggest that the lactose-free formulae may still exceed a carbohydrate load threshold for intestinal absorption, which may limit their usefulness in the context of complicated SAM.

**Trial registration:**

ClinicalTrials.gov NCT02246296.

## Abstract 2

Children with severe acute malnutrition (SAM) have high risk of inpatient mortality. In this trial, Robert H. J. Bandsma and colleagues test the hypothesis that a lactose-free, low-carbohydrate F75 milk might reduce these risks.

## Introduction

Children with complicated severe acute malnutrition (SAM) are admitted to the hospital because they are severely ill or unable to feed sufficiently [1]. In African hospitals, their risk of inpatient death ranges between 10%–30% [2–4]. In contrast, children with SAM who are clinically stable, i.e., without signs of illness and who have an appetite (uncomplicated SAM), are usually treated in community-based programs and have a substantially lower mortality risk, ranging between <1%–7% [5–7]. In hospital settings, mortality may be reduced to some extent by adhering to World Health Organization (WHO)–recommended management [2,4,8,9], but this may not address the full spectrum of infections and metabolic abnormalities of these seriously ill children [10–12]. In the 2013 Lancet Maternal and Child Nutrition series, improving management of SAM was identified as having the greatest likely impact on child mortality amongst nutritional interventions [13].

Current guidelines for the nutritional management of SAM in the hospital define 3 phases of treatment [14]: 1) the “stabilization phase,” during which children are fed a liquid diet (standard F75 [F75]) with a relatively low-protein (approximately 9 g/l) and relatively low-energy content (75 kcal/100 ml). F75 was designed to meet the estimated nutritional requirements to restore physiological and metabolic functions and to prevent refeeding syndrome while medical conditions stabilize. No weight gain is expected during this phase of treatment; 2) the “transition phase,” during which higher protein and energy through either F100 formula or ready-to-use therapeutic foods (RUTFs) are started with supplemental F75 formula; and 3) the “rehabilitation phase,” with an increased daily intake of F100 or RUTFs in order to achieve catch-up growth. Once a child has stabilized and tolerates RUTFs, WHO guidelines recommend discharge from hospital care, with continuation of the rehabilitation phase continued in the community [14,15].

The original F75 formulation was designed based on what was known about the pathophysiology of SAM at that time, before separation of treatment of complicated and uncomplicated SAM [16]. Today’s inpatient children with complicated SAM have a very different clinical profile from uncomplicated SAM treated in the community. Around 65% of calories in the F75 formula is derived from carbohydrates (maltodextrin, lactose, and sucrose). Once ingested, disaccharides such as lactose or sucrose are hydrolyzed into monosaccharides by disaccharidases found at the tip of small intestinal villi. Released monosaccharides such as glucose and galactose are then transported across the apical membrane through Na^+^-dependent glucose transporters, whereas fructose is taken up through a facilitative fructose transporter [17]. However, evidence suggests that children with SAM have impaired absorption of mono- and disaccharides, regardless of the presence of gastroenteritis [18,19]. Limited histological evidence also shows intestinal villous atrophy [18], which is consistent with clinical signs suggestive of carbohydrate malabsorption [20]. Thus, we hypothesized that revising the current F75 into a modified, lactose-free, and reduced-carbohydrate F75 formulation could reduce osmotic diarrhea and thereby lower the number of days to complete the first phase of treatment.

Early deterioration in children with SAM may also be related to refeeding syndrome, comprising severe metabolic derangements driven by insulin excretion after a sudden shift from a catabolic to an anabolic state [21]. Refeeding syndrome is characterized by hypophosphatemia, hypokalemia, and hypomagnesemia, which may impair cardiac, pulmonary, and neurological function and can result in (sudden) death. As protein synthesis is stimulated in anabolism, increased production of adenosine triphosphate (ATP) leads to a higher cellular demand for phosphate [21]. Furthermore, pancreatic insulin secretion induces the cellular uptake of glucose and electrolytes, including phosphate and potassium, and hypophosphatemia is common during nutritional rehabilitation of malnourished children and is associated with mortality [22–24]. We also hypothesized that a reduction in carbohydrate content of the modified F75 formula could lower the risk of refeeding syndrome. Together, reformulated F75 could plausibly improve early clinical outcomes among hospitalized children with SAM.

We conducted a randomized, double-blind controlled trial evaluating a modified F75 formula with substantially reduced carbohydrate content and without lactose versus the currently recommended formulation of F75 among hospitalized children with SAM in Kenya and Malawi.

## Methods

### Study design and participants

The study was a randomized, double-blind controlled trial conducted at two Kenyan hospitals (Kilifi County Hospital and Coast General Hospital, Mombasa) and one Malawian hospital (Queen Elizabeth Central Hospital, Blantyre). All children admitted to these study hospitals were screened for complicated SAM. The study was discussed in detail with parents or carers of potential participants in their local language, and written informed consent was sought. In patients for which re-establishing feeding was urgent, initial verbal assent was sought and followed up with written consent after treatment initiation.

Inclusion criteria were

- age 6 months to 13 years;
- SAM defined as mid-upper arm circumference (MUAC) < 11.5 cm or weight-for-height Z score < −3 if younger than 5 years of age, BMI Z score < −3 if older than 5 years, or edematous malnutrition at any age;
- being admitted to the hospital because of medical complications or failing an appetite test, as defined by WHO guidelines [16];
- weight-for-length/height or BMI, weight-for-age, and length/height-for-age Z scores were calculated using WHO 2006 and 2007 references.

Exclusion criteria were

- lack of informed consent,
- known allergy to milk products

### Investigational product

We compared F75 to the modified F75 (mF75) formulation (Table 1). Osmolarity was 298 mOsm/L and 232 mOsm/L for F75 and mF75, respectively. The formulations were isocaloric, and the reduced energy from carbohydrates was compensated by increased medium-chain triglycerides (MCTs), while protein and micronutrient composition were unaltered. Both formulations were manufactured by Nutriset (Nutriset, Malaunay, France) and dispensed by trained staff according to WHO recommendations: 95 kcal/kg per day, divided into 8 feeds per 24 hours.

**Table 1:** Formulations of F75 and mF75.

	% Energy		Quantity: g/1,000 ml	
**Macronutrients**	**F75**	**mF75**	**F75**	**mF75**
Protein	5.3%	5.3%	9.9	9.9
Lipid	31.5%	51.7%	26.3	43.1
Carbohydrate	63.2%	43.0%	118.5	80.6
Total	100%	100%	154.7	133.6
**Carbohydrate composition**				
Lactose	9.9%	0%	18.6	0
Sucrose	3.6%	3.6%	6.8	6.8
Maltodextrin	50%	39%	93.2	73.9

Abbreviations: F75, standard F75; mF75, modified F75.

### Randomization and masking

Sequential study numbers were computer generated using Stata Statistical Software: Release 12 (College Station, TX: StataCorp LP) with random block sizes for each site prior to the trial. An independent statistician was responsible for computerized sequence generation. Children were allocated study numbers sequentially at each site using sealed numbered envelopes. Group allocation was blinded to participants and all trial personnel, including trial coordinators and the principal investigator. The study products were identical in appearance and packaged in color-coded sachets.

### Procedures

Children were managed in the hospital as per Malawian or Kenyan national guidelines, both of which are based on WHO recommendations [15,16]. HIV testing by rapid antibody test was offered to all participants according to national guidelines, with appropriate counseling, follow-up tests, and referrals offered depending on results. Children were examined daily, and clinical status was recorded on a standardized proforma during ward rounds. If a child deteriorated after stabilization, they were returned to the stabilization phase and received F75 or mF75 as originally allocated. Blood and fecal samples were collected at admission and after 3 days of hospitalization.

The clinical team was blinded for the allocated formula and decided whether a child was fit for discharge based on criteria such as being in rehabilitation phase, clinical condition, ability to finish RUTF, and appetite level. RUTF, nutritional counseling, and follow-up in a nutrition clinic were provided at discharge, as per standard of care at each institution.

### Primary and secondary outcomes

The primary outcome was based on the purpose of F75, which is clinical stabilization, after which a child can progress to higher protein and energy feeds to promote catch-up growth. We therefore chose the number of days between admission and first stabilization as the primary outcome, defined as having reached the transition phase of treatment and switched to another feed type. Stabilization was based on the WHO guideline [15]:

1. absence of any WHO “danger” or emergency signs: obstructed breathing, respiratory distress, cyanosis, shock (delayed capillary refill plus fast and weak pulse plus temperature gradient), severe anemia (Hb < 5 g/dl), congestive cardiac failure, impaired consciousness, convulsions, severe dehydration, profuse watery diarrhea or vomiting, hypothermia;
2. loss of edema (if present on admission), defined as improving from a severe +++ edema (severe: generalized bilateral pitting edema) to ++ edema (moderate: no upper arm or upper leg edema and no facial edema), or from ++ edema to + edema (mild: only feet/ankle edema), or none;
3. and tolerating the full prescribed volume of F75 feeds and observed to be completing the feeds.

Study clinicians received additional training on the WHO guidelines as well as bedside and scenario-based training on the criteria for transition to improve standardization across sites. In order to include children who died before stabilization in the analyses, they were classified as “not stabilized.”

Although there is some subjectivity in determining a child’s medical condition that is “improving,” we chose stabilization as a main outcome rather than episodes of diarrhea because 1) it is the purpose of F75; 2) once a child switches to F100 or RUTFs, earlier transition could conceivably result in an increase in diarrhea unrelated to mF75 or F75; 3) diarrhea is very difficult to quantify objectively since maternal recall is not accurate in identifying episodes or severity [25]; 4) diapers may not distinguish every stool episode, and their weight is affected by urine; and 5) refeeding syndrome may be critical to early outcomes.

Prespecified secondary outcomes were the total number of days and number of days prior to stabilization with

1. diarrhea (i.e., 3 or more loose or watery stools within 24 hours),
2. receipt of rehydration fluids (ReSoMal or IV fluids),
3. signs of shock (i.e., fast and weak pulse with limb versus core temperature gradient and capillary refill time > 3 seconds),
4. lower chest wall indrawing,
5. hypoxemia (i.e., fingertip SaO_2_ < 90% or requiring oxygen to maintain SaO_2_ above 90%),
6. impaired consciousness (i.e., Blantyre coma score < 4) [26],
7. symptomatic hypoglycemia (i.e., blood sugar < 3.0 mmol/l, tested only on clinical indication),
8. vomiting,
9. congestive cardiac failure,
10. any antibiotics and second- or third-line antibiotics,
11. receiving F75 (initial stabilization plus any restabilization),
12. Nasogastric tube feeds or nonstandard feeds,
13. receiving breastmilk.

Other prespecified secondary outcomes were

1. mortality in hospital;
2. time to discharge from hospital in days;
3. the total number of days spent in the stabilization phase, including periods when children deteriorated and returned to the stabilization phase;
4. percentage change in weight between admission and day 5;
5. serum sodium, potassium, magnesium, calcium, phosphate, and albumin at day 3 and changes between admission and day 3;
6. stool osmotic gap and pH at day 3.

### Laboratory analyses

Blood was collected at admission and day 3, from which serum was obtained and stored at −80 °C. For analysis, samples were thawed overnight at 4 °C, vortexed, and centrifuged at 1,500 g for 3 minutes. Albumin, alanine aminotransferase (ALT), creatinine, calcium, magnesium, potassium, phosphate, sodium, and urea were batch analyzed using the P-module on a Roche Modular (Roche, Indianapolis, United States) at a single laboratory (University Medical Centre Groningen, the Netherlands). Fecal samples were collected at both time points and stored at −80 °C. Fecal biochemistry was batch analyzed only amongst patients with clinical diarrhea in the same laboratory. These samples were thawed overnight at 4 °C and weighed, diluted 3.5 times with distilled water, homogenized (Precellys 24 Homogenizer, Bertin, Rockeville, USA), and centrifuged at 16,100 g/rcf for 5 minutes. Fecal water was extracted, and osmolality was measured by freezing point depression (Osmo Station OM-6050, Arkray, Tokyo, Japan). In addition, sodium, potassium, and chloride were analyzed using the ISE900-module on Cobas 8000 (Roche, Indianapolis, USA). pH was determined using a standard pH meter. The stool osmotic gap was calculated by the equation 290 − 2 × (stool Na + stool K).

### Statistical analyses

A statistical analysis plan was reviewed and approved by an independent Data and Safety Monitoring Committee (DSMC) before the treatment allocation was unmasked, and the research database was locked. The DSMC oversaw the trial and reported to the Trial Steering Committee (TSC). An interim unblinded analysis was conducted by the DSMC during the trial after 50% of participants were recruited. A recommendation to the sponsor to discontinue recruitment, in all patients or in selected subgroups, would be made by the TSC on advice from the DSMC if the data provide proof beyond reasonable doubt that one of the treatment arms is better in terms of the primary outcome or safety guided by the Haybittle–Peto criteria. The Haybittle–Peto boundary states that if an interim analysis shows a probability of less than 0.001 that the treatments are different, then the trial should be stopped early.

For the primary endpoint of time to first stabilization, we regarded it as essential to include children who died before stabilization in the analysis. Therefore, the time to stabilization of children who died was entered as “999,” ensuring that they ranked bottom in the rank sum test and were included in the longest quartile of the IQR. An intention-to-treat analysis was conducted including all available data, including from children given the wrong formula at one or more feeds (primary endpoint evaluable) and children who withdrew or absconded, i.e., left the hospital without notifying the medical team or were transferred prior to stabilization (primary endpoint nonevaluable). We calculated the median (IQR) time to stabilization in the 2 randomized groups and compared their distributions using a Wilcoxon rank sum test and incidence rate ratio using Cox regression. We also conducted prespecified subgroup analyses to assess effect modification by site, HIV antibody status, edema status, and age using likelihood ratio tests. In addition, using the cmprsk R-package, we conducted competitive risk analysis to compare the cumulative incidence functions of the mutually exclusive risks of either dying prior to stabilization or achieving stabilization. Differences in these incidence functions indicate whether the cumulative probability of either dying before stabilization or achieving stabilization differs between groups as treatment progresses.

For secondary outcomes, we compared the number of days with prespecified clinical features between allocated groups using regression with a zero-inflated negative binomial distribution, since many children had no days with these problems. To compare the distributions of continuous variables, we used Student *t* test or Wilcoxon’s rank sum test if the variables were skewed. We compared categorical variables between randomized groups using χ^2^ or Fisher’s exact tests. Trends in proportions across ordered groups were assessed by a nonparametric test for trend. HIV test results were analyzed with “not done” as a separate category, as some children who died before testing or whose parents refused testing may have been more likely to have HIV infection or exposure. Blood and stool biochemical variables between intervention arms were compared while adjusting for site, whereas models comparing admission and day 3 included a random intercept for patients. All statistical analyses were done using either Stata version 13.0 (StataCorp 2013, Stata Statistical Software: Release 13; College Station, TX: StataCorp LP) or R statistical software version 3.4.0 [27].

### Sample size

Based on prior trial experience [28], we estimated that 50% of children allocated to F75 would stabilize by day 5, and we calculated the sample size required to demonstrate a 10% absolute increase in percentage stabilized at day 5 to 60% in the intervention arm (equivalent to a hazard ratio of 1.35). With an alpha of 5% and power of 80%, the number of patients required was 381 per arm, and to allow for 10% anticipated losses, we aimed to recruit 420 per arm.

### Ethical considerations

The trial was registered at ClinicalTrials.gov, number NCT02246296. Ethical approval was obtained from the College of Medicine Research Ethics Boards of the University of Malawi (P.03/14/1540), the KEMRI Ethical Review Committee (SCC 2799), the Oxford Tropical Research Ethics Committee (OXTREC 58–14), and the Hospital for Sick Children Research Ethics Board, Toronto (1000046559).

## Results

### Patient characteristics

**Fig 1:**
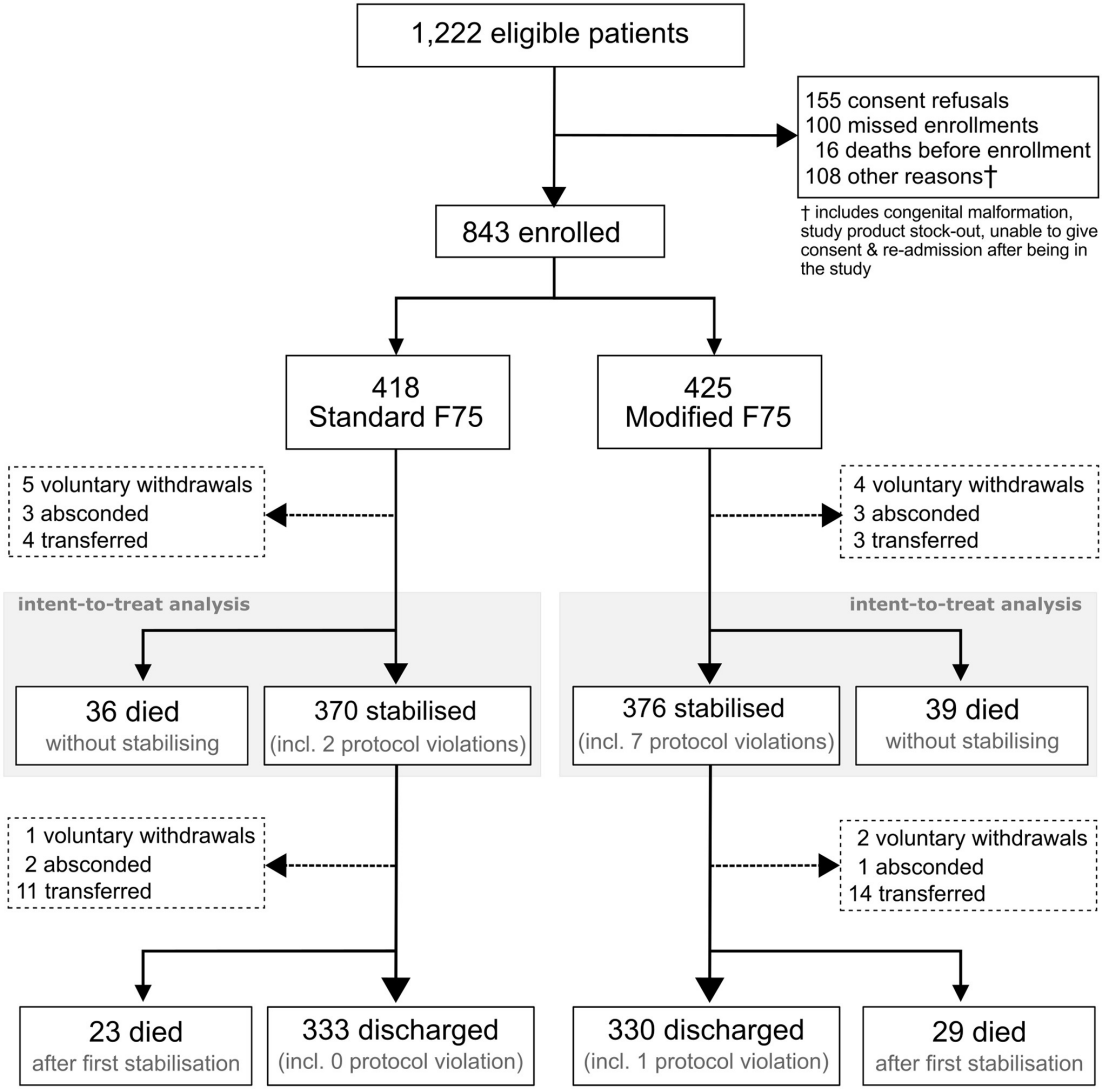
Numbers of children who were screened, assigned a trial group, and included in the primary analysis. Children aged 6 months to 13 years who were admitted for complicated SAM were screened for eligibility in the participating hospitals. They were enrolled after informed consent was obtained. SAM, severe acute malnutrition.

Between 4 December 2014 and 24 December 2015, 843 hospitalized children were enrolled, 418 were allocated to F75, and 425 were allocated to mF75 (Fig 1). The median age of participants was 16 months (IQR 10 to 25 months); 385 (46%) were female, 264 (31%) had kwashiorkor, 382 (45%) had diarrhea at admission, and 179 (21%) had a positive HIV test, with 46 (5.5%) having a declined or missed HIV test. Overall, 516 (61%) were enrolled in Kenya and 327 (39%) in Malawi. Baseline clinical and demographic characteristics were similar between randomized groups and are presented in Table 2.

**Table 2:** Baseline characteristics.

	F75	mF75
Demographics	(*n* = 418)	(*n* = 425)
**Age in months (median, IQR)**	16 (10–26)	16 (10–25)
**Female**	180 (43)	205 (48)
**Primary caregiver is mother**	388 (93)	402 (95)
**Site**		
**Kilifi County Hospital, Kenya**	92 (22)	95 (22)
**Coast General Hospital, Kenya**	164 (39)	165 (39)
**Queen Elizabeth Central Hospital, Malawi**	162 (39)	165 (39)
**Nutrition**		
**MUAC (mean, SD)**	11.2 (1.4)	11.3 (1.5)
Weight-for-age Z score* **(mean, SD)**	−4.1 (1.2)	−4.0 (1.1)
Weight-for-height/length Z score* **(mean, SD)**	−3.8 (1.1)	−3.7 (1.0)
BMI Z score* **(mean, SD)**	−4.7 (1.7)	−3.9 (1.3)
**Height/length-for-age Z score (mean, SD)**	−2.8 (1.8)	−2.8 (1.7)
**Edema**	129 (31)	135 (32)
**Currently breastfeeding**	186 (45)	205 (48)
**Clinical condition at admission**		
**Signs of shock**	17 (4.1)	9 (2.1)
**Impaired consciousness**	14 (3.4)	16 (3.8)
**Severe pneumonia**	106 (25)	105 (25)
**Diarrhea**	194 (46)	188 (44)
**Temperature ≥ 38.5 °C**	121 (29)	123 (29)
**Temperature < 36.5 °C**	28 (6.7)	19 (4.5)
**Oxygen or SaO_2_ < 90%**	31 (7.4)	34 (8.0)
**Malaria parasitemia**	29 (7.0)	38 (8.9)
**Chronic conditions**		
**Tuberculosis**	12 (2.9)	6 (1.4)
**Cerebral palsy**	62 (15)	68 (16)
**Chronic cough**	28 (6.7)	24 (5.7)
**Sickle cell disease**	1 (0.2)	1 (0.2)
**Congenital or acquired heart disease**	10 (2.4)	7 (1.7)
**HIV antibody test**		
**Positive**	98 (23)	81 (19)
**Untested**	18 (4.3)	28 (6.6)

Abbreviations: F75, standard F75; mF75, modified F75; MUAC, mid-upper arm circumference.

All data are *n* (%) unless otherwise indicated.

*Excludes children with edema. WAZ, weight-for-age z scores; WHZ, weight-for-height z scores; and HAZ, height-for-age z scores are calculated for children <5 years old.

Of a total of 7,098 child days of hospitalization, 2,615 were prior to stabilization, and 75/843 (8.9%) children died prior to stabilization. Nine children voluntarily withdrew from the study prior to stabilization, and 6 absconded from the hospital (as defined above). Seven children were transferred to another hospital, 4 of whom because of hospital closure due to a health worker strike. Nine children (1.0%) received the wrong formula at one or more feeding times and were included in the primary analysis.

### Primary outcome

**Fig 2:**
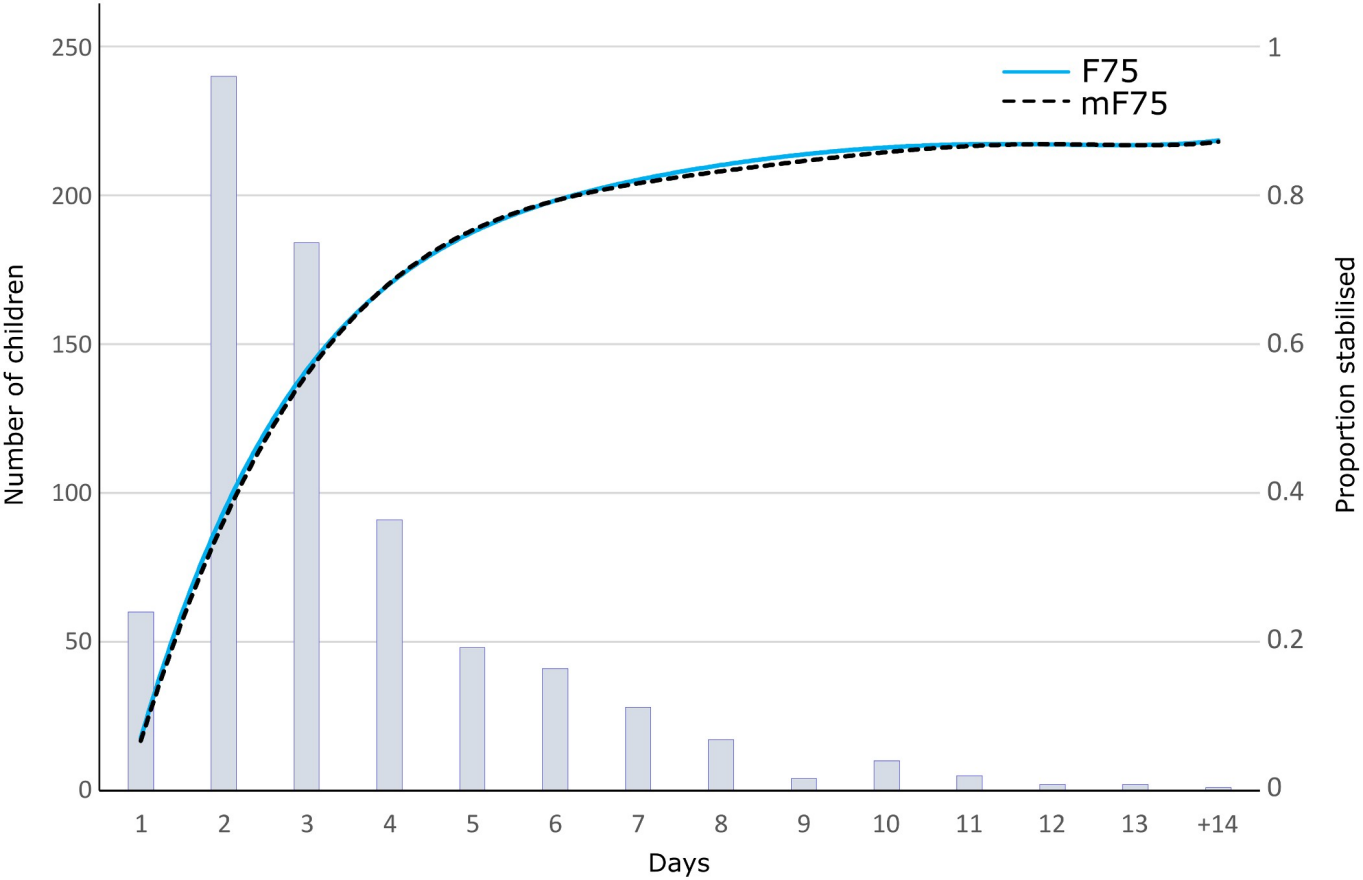
Primary endpoint, time to stabilization. Blue bars indicate the number of children who stabilized each day; the cumulative proportion stabilized allocated to F75 is shown by the blue line and allocated to mF75 by the dashed black line. F75, standard F75; mF75, modified F75.

The median time to stabilization was 3 days (IQR 2–6 days) (Fig 2, Table 3, and S1 Fig), and there was no evidence for difference in time to stabilization between randomized groups; absolute difference 0.23 (95% CI −0.13 to 0.60, *P* = 0.59). Prespecified subgroup analyses did not suggest effect modification by the presence of diarrhea at admission, age, edema, or site (S1 Table). There was evidence of an effect of HIV status (P < 0.0001) on both time to stabilization and time to mortality but no interaction between HIV status and effect of allocated feeds (*P* ≥ 0.33, S2 Table).

**Table 3:** Primary and secondary outcomes.

Primary outcome	F75 (*n* = 418)	mF75 (*n* = 425)	IRR (95% CI)	*P* value
Days to stabilization, median, IQR^#^	3 (2–5)	3 (2–5)	1.05 (0.91, 1.22)	0.48
**Secondary outcomes prior to first stabilization**	1,349 child days	1,266 child days		
Children who died, *n* (%)	36 (8.6)	39 (9.0)	0.97 (0.62, 1.52)	0.89
Children having a severe adverse event, *n* (%)	57 (14)	60 (14)	1.02 (0.65, 1.60)	0.92
	Days	Days		
Diarrhea	521	469	1.03 (0.81, 1.30)	0.84
Vomiting	247	275	1.36 (0.94, 1.95)	0.10
ReSoMal treatment	593	555	0.98 (0.80, 1.24)	0.98
IV fluid treatment	71	50	0.70 (0.29, 1.70)	0.43
Signs of shock	32	19	0.79 (0.11, 1.69)	0.08
Oxygen therapy	110	70	0.87 (0.48, 1.58)	0.65
Impaired consciousness	67	58	1.03 (0.51, 2.08)	0.94
Symptomatic hypoglycemia	11	6	0.17 (0.01, 2.83)	0.22
Receiving antibiotics	1,106	1,060	0.96 (0.84, 1.09)	0.51
Receiving 2nd line antibiotics	366	294	0.83 (0.59, 1.17)	0.28
Receiving nonstandard feeds	55	36	0.58 (0.34, 1.02)	0.06
Nasogastric tube in situ	238	230	1.02 (0.72, 1.44)	0.92
Subcostal chest wall indrawing	183	159	0.86 (0.55, 1.36)	0.52
Congestive cardiac failure	25	9	0.17 (0.05, 0.61)	0.007
Receiving breastmilk	490	443	0.78 (0.61, 0.98)	0.04
**Secondary outcomes during hospitalization**	3,546 child days	3,553 child days		
Death, *n* (%)	59 (14)	68 (16)	1.20 (0.82, 1.76)	0.34
	Days	Days		
Days of hospitalization in survivors, median, IQR	8 (6–11)	8 (6–12)	1.02 (0.93, 1.12)	0.73
Days from stabilization to discharge, median, IQR	7 (5–11)	7 (5–12)	1.05 (0.94, 1.17)	0.39
Days in stabilization, including restabilization	1,610	1,650	1.09 (0.82, 1.44)	0.55
Receiving antibiotics	3,145	3,096	0.98 (0.90, 1.07)	0.70
Receiving 2nd line antibiotics	1,359	1,245	0.94 (0.80, 1.10)	0.44
Receiving nonstandard feeds	176	134	0.64 (0.38, 1.07)	0.09
Nasogastric tube in situ	345	329	1.10 (0.80, 1.51)	0.55

Abbreviations: F75, standard F75; IRR, incidence rate ratio; mF75, modified F75

Except where otherwise indicated, all data are the total number of days in which the children experienced clinical signs, and this included children who subsequently died the counts of days with clinical signs. IRRs were obtained from site-adjusted zero-inflated negative binomial regression models.

^#^Differences in days to stabilization between F75 and mF75 were tested using hazard ratio from a Cox regression model adjusted for site. *P* values are unadjusted for multiple comparison.

### Secondary clinical outcomes

There was no effect of the intervention on mortality before stabilization (*P* = 0.84) or during hospitalization overall (*P* = 0.44, Table 3). As presented in S3 Table, 117 (14%) children experienced a serious adverse event before stabilization without evidence of difference between randomized groups (*P* = 0.59). Diarrhea was common; of the 416 children without diarrhea at admission, 176 (38%) developed diarrhea in hospital. The number of days with diarrhea prior to stabilization and during the whole hospitalization period were similar in both groups (Table 3). There were no significant differences in the number of days with vomiting or receiving rehydration fluids (ReSoMal or IV fluids). The types of other feeds children received (nonstandard milks) and routes (nasogastric or oral) were not affected by randomized allocation, except breast milk, which was lower in the mF75 group (*P* = 0.04). Hypoglycemia (<3.0 mmol/l) rarely occurred, and the number of days with an episode of hypoglycemia was not influenced by the intervention (Table 3). The number of days receiving antibiotics was also not affected by the intervention and neither was the mean (±SD) percent of weight change between admission and day 5 (3.1% ± 5.8% in nonoedematous, *P* = 0.78; −3.4% ± 7.1% in oedematous patients, *P* = 0.63). Although uncommonly diagnosed, the incidence of clinician-defined congestive heart failure was lower in the mF75 group (*P* = 0.007).

### Biochemical outcomes

Table 4 shows the serum and fecal biochemistry. There were no differences between the overall distributions of biochemical concentrations in serum between the standard and mF75 on day 3. Hyponatremia was common, both at admission and after 3 days (Table 5), without differences between the groups, whereas hypernatremia was rare. The prevalence of abnormal potassium concentrations was not different between the groups. Hypokalemia was frequently found but decreased in prevalence between admission and day 3, whereas hyperkalemia increased. Hypermagnesemia was more common than hypomagnesaemia at admission, but not on day 3, and no differences were found between the F75 and mF75 group. Hypophosphatemia was present in 10% of children at admission, declining to 5.9% at day 3, with higher prevalence in the mF75 group (*P* = 0.009).

**Table 4:** Biochemical differences in serum and stool between patients receiving F75 or mF75 formula.

					Admission versus Day 3				F75 versus mF75			
Serum, median (IQR)	All Admission	All Day 3	F75 Day 3	mF75 Day 3	β	95% CI	df	*P*	β	95% CI	df	*P*
ALT, U/L	23 (14–44)	29 (18–53)	28 (18–53)	30 (18–53)	0.68	[−13 to 6.1]	579	0.47	6.2	(−44 to 20)	662	0.47
Albumin, g/L	34 (24–41)	32 (23–38)	32 (22–38)	32 (24–39)	−0.77	[−1.1 to −0.43]	538	<0.0001	0.79	(−0.61 to 2.2)	633	0.27
Calcium, mmol/L	2.2 (2–2.3)	2.2 (2–2.3)	2.2 (2–2.3)	2.2 (2–2.3)	0.025	[−0.000044 to 0.050]	638	0.051	0.023	(−0.025 to 0.071)	634	0.35
Magnesium, mmol/L	0.87 (0.79–0.97)	0.86 (0.77–0.95)	0.85 (0.74–0.93)	0.87 (0.77–0.96)	−0.036	[−0.052 to −0.020]	672	<0.0001	0.026	(−0.00096 to 0.052)	614	0.059
Phosphate, mmol/L	1.3 (0.98–1.6)	1.5 (1.1–1.8)	1.6 (1.2–1.9)	1.5 (1.1–1.8)	0.20	[0.15 to 0.24]	555	<0.0001	−0.087	(−0.18 to 0.0072)	603	0.071
Potassium, mmol/L	4.5 (3.5–5.2)	5.3 (4.7–6.1)	5.2 (4.7–6)	5.4 (4.8–6.4)	1.8	[0.96 to 2.6]	822	<0.0001	−0.12	(−1.6 to 1.4)	685	0.88
Sodium, mmol/L	136 (132–139)	135 (132–138)	135 (132–138)	136 (132–139)	−0.22	[−0.80 to 0.34]	723	0.43	0.041	(−0.79 to 0.88)	685	0.92
Creatinine, μmol/L	24 (19–32)	22 (17–28)	22 (17–28)	22 (17–29)	−3.6	[−5.5 to −1.8]	633	0.0002	2.6	(−0.44 to 5.7)	623	0.094
Urea, mmol/L	2.7 (2–3.9)	2 (1.3–2.9)	2 (1.4–2.9)	2 (1.2–3)	−1.0	[−1.3 to −0.79]	665	<0.0001	−0.028	(−0.35 to 0.29)	608	0.86
**Fecal, median (IQR)**												
Chloride, mmol/kg	19 (15–30)	17 (14–22)	17 (14–23)	18 (14–22)	-	-	-	-	−0.43	(−4.1 to 3.3)	80	0.82
Potassium, mmol/kg	47 (36–63)	54 (42–67)	55 (43–67)	52 (42–72)	-	-	-	-	2.7	(−5.5 to 11)	81	0.52
Sodium, mmol/kg	21 (7–42)	4 (0–10)	5.5 (0–14)	0 (0–10)	-	-	-	-	−3.9	(−9.3 to 1.4)	80	0.15
Osmolarity, mOsmol/kg	354 (315–416)	357 (318–392)	356 (315–391)	357 (332–4 06)	-	-	-	-	28	(−0.84 to 57)	89	0.060

Abbreviations: ALT, alanine aminotransferase; CI, confidence interval; df, degrees of freedom; F75, standard F75; mF75, modified F75.

Models comparing blood and stool biochemistry variables between groups at day 3 were adjusted for site, whereas a random intercept for patient was included when comparing timepoints.

*Fecal biochemistry was only analyzed in children with clinical diarrhea, and comparison of admission with day 3 was not performed because of the small number and likely high selection bias of samples when comparing admission to day 3. Coefficients (β) and 95% confidence intervals express concentration differences between comparison groups; the reference groups were as appropriate children at admission or those receiving standard F75 formula. *P* values are unadjusted for multiple comparison, significance threshold, *P* < 0.05.

**Table 5:** Prevalence of abnormal blood biochemistry at admission and day 3 in patients receiving F75 or mF75 formula.

	All Admissions	F75	mF75	*P*	All Day 3	F75	mF75	*P*
	*n* = 683	*n* = 318	*n* = 322		*n* = 689	*n* = 340	*n* = 349	
Low								
Sodium, <135 mmol/L	278 (41%)	124 (37%)	154 (45%)	0.036	294 (43%)	152 (45%)	142 (41%)	0.32
Potassium, <3.5 mmol/L	158 (23%)	75 (22%)	83 (24%)	0.59	32 (4.6%)	12 (3.5%)	20 (5.7%)	0.21
Magnesium, <0.7 mmol/L	66 (10%)	26 (8.2%)	40 (12%)	0.091	93 (15%)	51 (17%)	42 (13%)	0.26
Phosphate, <0.7 mmol/L	63 (10%)	27 (8.7%)	36 (11%)	0.29	36 (5.9%)	8 (2.6%)	28 (9.2%)	0.0009
High								
Sodium, >145 mmol/L	25 (3.7%)	13 (3.8%)	12 (3.5%)	0.84	20 (2.9%)	10 (2.9%)	10 (2.9%)	1.0
Potassium, >5 mmol/L	202 (30%)	98 (29%)	104 (30%)	0.80	417 (61%)	191 (56%)	226 (65%)	0.024
Magnesium, >1 mmol/L	120 (19%)	55 (17%)	65 (20%)	0.36	83 (13%)	30 (9.8%)	53 (17%)	0.013
Phosphate, >1.5 mmol/L	190 (30%)	97 (31%)	93 (29%)	0.66	317 (52%)	160 (53%)	157 (51%)	0.75

Abbreviations: F75, standard F75; FDR, false discovery rate; mF75, modified F75.

Data are presented as *n* (%) of patients with blood electrolyte values above or below normal ranges. Differences between comparison groups in the prevalence of abnormal values were tested using Fisher’s exact test, and both uncorrected and false discovery rate (FDR) corrected *P* values are presented. Missing electrolyte values are as follows: at admission: potassium, *n* = 13; magnesium, *n* = 45; phosphate, *n* = 52; at day 3: potassium, *n* = 98; magnesium, *n* = 77; phosphate, *n* = 81. *P* values are unadjusted for multiple comparison, significance threshold, *P* < 0.05.

Among children with diarrhea on day 3, when stool collection was undertaken, 88% had a fecal osmotic gap >100 mOsm/kg, indicating osmotic diarrhea (Table 6). There were no differences between the randomized groups in the prevalence of low <50 mOsm/kg or high >100 mOsm/kg stool osmotic gap at day 3.

**Table 6:** Prevalence of low and high stool osmotic gap at admission or day 3 in patients receiving F75 or mF75 formula that had signs of clinical diarrhea.

	All Admission	F75	mF75	*P*	All Day 3	F75	mF75	*P*
Osmotic Gap	*n* = 112	*n* = 59	*n* = 53		*n* = 84	*n* = 52	*n* = 32	
Low, <50	7 (6.3%)	3 (5.1%)	4 (7.5%)	0.71	1 (6.3%)	1 (1.9%)	0 (0%)	1.0
High, >100	80 (71%)	38 (64%)	42 (79%)	0.096	74 (88%)	47 (90%)	27 (84%)	0.49

Abbreviations: F75, standard F75; FDR, false discovery rate; mF75, modified F75.

Data are presented as *n* (%) of patients with diarrhea for which calculated stool osmotic gap values were above or below normal ranges. Differences between comparison groups in the prevalence of abnormal values were tested using Fisher’s exact test, and both uncorrected and FDR corrected *P* values are presented.

## Discussion

We tested a significantly modified formulation of F75 among hospitalized children with SAM. There was no evidence of effect on the primary outcome of time to stabilization. The median time to stabilization was 3 days, which was shorter than anticipated, possibly due to careful provision and timing of F75 feeds but within the range suggested by WHO guidelines [14]. Combined with the fact that there was also no evidence of differences in any of the secondary outcomes, we conclude that an approximately 30% reduction in carbohydrate load without lactose is not more effective than standard F75. Similarity in the primary and secondary outcomes between groups does not suggest that the result was due to a lack of statistical power. The overall inpatient case fatality ratio of 15% was similar to estimates reported before by the participating sites [12,28].

There is strong evidence from observational studies that carbohydrate digestion and absorption is impaired in severely malnourished children [18,19]. Consequently, ingesting carbohydrates beyond the intestinal capacity for absorption can lead to osmotic diarrhea. Diarrhea has been associated with mortality in severely malnourished children [29,30], and cases of osmotic diarrhea often present with clinical complications of electrolyte disturbances and dehydration. However, there is limited evidence from intervention studies. Among children with SAM, Kerpel-Fronius and colleagues gave boluses of 2–2.5 g/kg of different types of carbohydrates and found that lactose administration especially lowered stool pH, a marker of carbohydrate malabsorption [31]. These sugar boluses were in a physiological range that is similar to a 3-hourly feed of F75 and suggested that changing lactose to sucrose might aid absorption. Kukurozovic and colleagues evaluated the effects of different lactose-free formulas in malnourished Australian aboriginal children presenting with diarrhea and found significant differences in the duration of diarrhea between the different formulas, potentially related to their osmolality [32]. The modification of energy from carbohydrates in our study was substantial (approximately 30% reduction) and suggests no linear relationship between the amount of carbohydrates ingested and stool frequency or consistency in this population. This finding is consistent with mF75 still exceeding a threshold for intestinal absorption among children who had diarrhea.

Most experience on feeding children with severely impaired intestinal function has been gained from treating short bowel syndrome, for which carbohydrates can be a limiting factor in the ability to advance feeds, thus use of complex carbohydrates is commonly advised [33]. The carbohydrates used in the F75 formula apart from lactose are, for the most part, maltodextrin, which is a complex carbohydrate but shorter and with more loosely bound links than starches. Limited evidence has suggested that starches might be beneficial in patients with short bowel syndrome, which could also be relevant for children with SAM [34]. However, a more radical reformulation of F75 would have to ensure supply of energy in a form that prevents hypoglycemia. We are aware of an ongoing study evaluating whether lactose-free formula affects the outcome of severely malnourished children (www.isrctn.com/ISRCTN98124592).

Diarrhea in SAM is multifactorial; infectious causes and intestinal inflammation as well as impaired digestion of fat and protein related to exocrine pancreas and hepatobiliary dysfunction likely impact the overall nutrient absorptive capacity, and these pathways would not be directly ameliorated by our intervention [12,35,36]. Most cases of diarrhea were osmotic in nature rather than secretory, suggesting that bacterial infections such as enterotoxin-producing *Escherichia coli* or rotavirus were likely not contributing substantially to the diarrhea at day 3. We did not determine diarrhea etiology to estimate the contribution of infectious causes.

Biochemical findings suggest that refeeding syndrome was relatively uncommon on day 3 in our study. The incidence of refeeding syndrome during treatment of severely malnourished children is not well established due to the lack of clear defining criteria and serial biochemical measurements. The prevalence of hypophosphatemia (<0.70 mmol/l) on day 3, an indicator of refeeding syndrome, was relatively low at 5.9% in our cohort but significantly higher in the mF75 group, although the median serum phosphate did not differ. We found no other differences in biochemical features of refeeding syndrome. Kimutai and colleagues reported an increase in hypophosphatemia (<1.20 mmol/l) from 6% on admission to 22% on the second day of hospitalization of children with SAM [37]. A recent study in Uganda reported hypophosphatemia on the second day of hospitalization in 58% of children with SAM who died and 13% in the children who survived [24]. The cutoff was much higher than in our study at 1.6 mmol/l for infants and 1.1 mmol/l in older children. In the latter study by Rytter and colleagues, children with diarrhea were given a rice porridge, which has a higher carbohydrate content than F75, and although not a trial, mortality was reported to be higher in this group. Another possible explanation for the low prevalence of severe biochemical signs of refeeding syndrome in SAM might also be related to the observation that insulin response is impaired in SAM children [38].

We choose to replace the carbohydrates with lipids, in particular with MCTs, for a number of reasons. First, MCTs are more rapidly hydrolyzed in the intestinal lumen than long-chain triglycerides, and MCTs do not require pancreatic lipase or bile for intestinal absorption. Secondly, MCTs are more easily absorbed, as they bypass the lymphatic system. MCTs require no or minimal carnitine to enter mitochondria, which could be beneficial if carnitine levels are deficient. Finally, lipid malabsorption is thought not to lead to dehydration or electrolyte disturbances. Despite these potential theoretical beneficial effects for critically ill malnourished children, our study did not suggest that increased MCTs impact overall clinical outcome.

Strengths of this trial are the study size; the double-blind and multicenter design, thereby enhancing its generalizability; and it being carried out in routine practice conditions. We captured detailed daily clinical information, which gave us unique insight into clinical evolution. There has been a long-standing debate on how to best transition children from the F75 formula to RUTFs or the F100, with similar concerns about increasing carbohydrate load and the risk of exacerbating or new onset diarrhea [15]. This study also directly informs this debate and suggests that reducing the carbohydrate content during transition or rehabilitation might not impact clinical outcome. There is one recent trial from our group that supports this, although more and larger trials are needed for generalizability [39]. The main limitation of the study is that the main outcome, stabilization with transition to the next phase of therapy, was based on WHO guidelines, comprising clinical evidence of recovery from acute illness as well as metabolic stabilization evidenced by recovery of appetite. We chose this main outcome as opposed to diarrhea for a number of reasons. Firstly, stabilization is the purpose of using F75 milk formula. Diarrhea is difficult to assess objectively, particularly in our setting. Maternal recall is not accurate in identifying diarrhea or assessing its severity [25]. Alternatively, the systematic observation of diapers could have been introduced; however, considering the added clinical load, the implementation in our setting was not feasible. For example, determining stool frequency would have required very frequent checks over 24-hour periods by already short-staffed research nurses. Importantly, the lack of improvement in individual daily clinical signs in our trial strongly suggests that the similar results obtained from the different formulations on the primary outcome is not due to subjective variation in the clinical decision of transitioning a child from the stabilization phase.

In conclusion, using a reduced-carbohydrate, lactose-free F75 did not improve the time to stabilization or other clinical outcomes of children with complicated severe malnutrition. The results support the ongoing use of the current F75 formulation and suggest that empiric use of lactose-free formula for children with diarrhea is not needed. It is important to explore whether our findings are replicated in an Asian context, as there could be Africa/Asia differences in SAM and associated infections. Finally, the carbohydrate content and composition are not the only determinants of F75 efficacy, and other aspects of treatment are also likely to be important.

## Supporting information

S1 CONSORT checklist (PDF)

S1 Protocol Randomized controlled trial of a reduced-carbohydrate formulation of F75 therapeutic milk among children with SAM. SAM, severe acute malnutrition.
(DOCX)

S1 Table Primary endpoint and prespecified subgroup analyses. All data are median (IQR) or geometric means (95% CI), as indicated.
(DOCX)

S2 Table Competitive risk models. All data are presented as median (IQR).
(DOCX)

S3 Table List of serious adverse events. (DOCX)

S1 Fig Probability of stabilization and death between the two treatment groups. Competing risk analysis simultaneously compares the estimated cumulative incidence curves of mutually exclusive events: first stabilization (solid line) or death prior to stabilization (dashed lines). Withdrawals and absconded cases were censored. Differences in cumulative incidence functions between F75 (blue line) and mF75 (black line) and all subgroup analysis models were compared using Gray’s test. Significance threshold, *P* < 0.05. F75, standard F75; mF75, modified F75.
(TIF)

## Abbreviations

ALT: alanine aminotransferase
ATP: adenosine tri-phosphate
df: degrees of freedom
DSMC: Data and Safety Monitoring Committee
F75: standard F75
FDR: false discovery rate
HAZ: height-for-age z scores
IRR: incidence-rate ratio
MCT: medium-chain triglyceride
mF75: modified F75
MUAC: mid-upper arm circumference
RUTF: ready-to-use therapeutic food
SAM: severe acute malnutrition
TSC: Trial Steering Committee
WAZ: weight-for-age z scores
WHO: World Health Organization
WHZ: weight-for-height z scores

## References

[1] Trehan I, Manary MJ. Management of severe acute malnutrition in low-income and middle-income countries. Arch Dis Child. 2015; 100(3):283–287. 10.1136/archdischild-2014-306026 25421910

[2] Ashworth A, Chopra M, McCoy D, Sanders D, Jackson D, Karaolis N, et al WHO guidelines for management of severe malnutrition in rural South African hospitals: Effect on case fatality and the influence of operational factors. Lancet. 2004(9415); 1110–1115. 10.1016/S0140-6736(04)15894-7 15064029

[3] Maitland K, Berkley JA, Shebbe M, Peshu N, English M, Newton CRJC. Children with severe malnutrition: Can those at highest risk of death be identified with the WHO protocol? PLoS Med. 2006; 3:2431–2439. 10.1371/journal.pmed.0030500 17194194 PMC1716191

[4] Deen JL, Funk M, Guevara VC, Saloojee H, Doe JY, Palmer A, et al Implementation of WHO guidelines on management of severe malnutrition in hospitals in Africa. Bull World Health Organ. 2003; 81(4):237–243. 12764489 PMC2572430

[5] Trehan I, Goldbach HS, LaGrone LN, Meuli GJ, Wang RJ, Maleta KM, et al Antibiotics as part of the management of severe acute malnutrition. N Engl J Med. 2013; 368(5):425–435. 10.1056/NEJMoa1202851 23363496 PMC3654668

[6] Alvarez Morán JL, Alé GBF, Charle P, Sessions N, Doumbia S, Guerrero S. The effectiveness of treatment for Severe Acute Malnutrition (SAM) delivered by community health workers compared to a traditional facility based model. BMC Health Serv Res. 2018; 18(1):207 10.1186/s12913-018-2987-z 29580238 PMC5870488

[7] Burza S, Mahajan R, Marino E, Sunyoto T, Shandilya C, Tabrez M, et al Community-based management of severe acute malnutrition in India: new evidence from Bihar. Am J Clin Nutr. 2015; 101(4):847–59. 10.3945/ajcn.114.093294 25833981 PMC4381773

[8] Collins S, Dent N, Binns P, Bahwere P, Sadler K, Hallam A. Management of severe acute malnutrition in children. Lancet. 2006; 368(9551):1992–2000. 10.1016/S0140-6736(06)69443-9 17141707

[9] Hossain M, Chisti MJ, Hossain MI, Mahfuz M, Islam MM, Ahmed T. Efficacy of World Health Organization guideline in facility-based reduction of mortality in severely malnourished children from low and middle income countries: A systematic review and meta-analysis. J Paediatr Child Health. 2017; 53:474–479. 10.1111/jpc.13443 28052519

[10] Heikens GT, Bunn J, Amadi B, Manary M, Chhagan M, Berkley JA, et al Case management of HIV-infected severely malnourished children: challenges in the area of highest prevalence. Lancet. 2008; 371:1305–1307. 10.1016/S0140-6736(08)60565-6 18406865

[11] Rytter MJ, Babirekere-Iriso E, Namusoke H, Christensen VB, Michaelsen KF, Ritz C, et al Risk factors for death in children during inpatient treatment of severe acute malnutrition: a prospective cohort study. Am J Clin Nutr. 2016; 105(2):494–502. ajcn140822. 10.3945/ajcn.116.140822 28031190

[12] Attia S, Versloot CJ, Voskuijl W, Van Vliet SJ, Di Giovanni V, Zhang L, et al Mortality in children with complicated severe acute malnutrition is related to intestinal and systemic inflammation: An observational cohort study. Am J Clin Nutr. 2016; 104:1441–1449. 10.3945/ajcn.116.130518 27655441 PMC5081715

[13] Bhutta ZA, Das JK, Rizvi A, Gaffey MF, Walker N, Horton S, et al Evidence-based interventions for improvement of maternal and child nutrition: what can be done and at what cost? Lancet. 2013; 382:452–477. 10.1016/S0140-6736(13)60996-4 23746776

[14] WHO. Pocket Book of Hospital Care for Children: Guidelines for the Management of Common Childhood Illnesses [Internet]. Guidelines for the management of common illnesses. 2013. 10.1016/j.cardfail.2011.02.010 24006557

[15] World Health Organization. updates on the management of severe acute malnutrition in infants and children. [Internet]. 2013. http://apps.who.int/iris/bitstream/10665/95584/1/9789241506328_eng.pdf Date of citation, May 18, 2018. 24649519

[16] Management of severe malnutrition: a manual for physicians and other senior health workers [Internet]. 1999. http://apps.who.int/iris/bitstream/10665/41999/1/a57361.pdf Date of citation, May 18, 2018.

[17] Wright EM, Martín MG, Turk E. Intestinal absorption in health and disease—sugars. Best Pract Res Clin Gastroenterol. 2003; 17:943–956. 10.1016/S1521-6918(03)00107-0 14642859

[18] Kvissberg MA, Dalvi PS, Kerac M, Voskuijl W, Berkley JA, Priebe MG, et al Carbohydrate malabsorption in acutely malnourished children and infants: A systematic review. Nutr Rev. 2016; 74:48–58. 10.1093/nutrit/nuv058 26578625 PMC4684688

[19] Bandsma RHJ, Spoelstra MN, Mari A, Mendel M, van Rheenen PF, Senga E, et al Impaired glucose absorption in children with severe malnutrition. J Pediatr. 2011; 158:282–287.e1. 10.1016/j.jpeds.2010.07.048 20843523

[20] Prinsloo JG, Wittmann W, Pretorius PJ, Kruger H, Fellingham SA. Effect of different sugars on diarrhoea of acute kwashiorkor. Arch Dis Child. 1969; 44:593–599. 10.1136/adc.44.237.593 5394500 PMC2020074

[21] Ormerod C, Farrer K, Harper L, Lal S. Refeeding syndrome: a clinical review. Br J Hosp Med. 2010;71:686–90. 21135765 10.12968/hmed.2010.71.12.686

[22] Yoshimatsu S, Hossain MI, Islam MM, Chisti MJ, Okada M, Kamoda T, et al Hypophosphatemia among severely malnourished children with sepsis in Bangladesh. Pediatr Int. 2013; 55:79–84. 10.1111/j.1442-200X.2012.03724.x 22978457

[23] Manary MJ, Hart CA, Whyte MP. Severe hypophosphatemia in children with kwashiorkor is associated with increased mortality. J Pediatr. 1998; 133:789–791. 10.1016/S0022-3476(98)70153-2 9842046

[24] Rytter MJ, Babirekere-Iriso E, Namusoke H, Christensen VB, Michaelsen KF, Ritz C, et al Risk factors for death in children during inpatient treatment of severe acute malnutrition: a prospective cohort study. Am J Clin Nutr. 2017; 105:494–502. 10.3945/ajcn.116.140822 28031190

[25] Voskuijl W, Potani I, Bandsma R, Baan A, White S, Bourdon C, et al Stool frequency recording in severe acute malnutrition ('StoolSAM’); An agreement study comparing maternal recall versus direct observation using diapers. BMC Pediatr. 2017; 17 10.1186/s12887-017-0874-0 28592288 PMC5461774

[26] Molyneux ME, Taylor TE, Wirima JJ, Borgstein A. Clinical features and prognostic indicators in paediatric cerebral malaria: A study of 131 comatose malawian children. QJM. 1989; 71:441–459. 10.1093/oxfordjournals.qjmed.a068338 2690177

[27] R: A language and environment for statistical computing. [Internet]. Vienna; 2013. http://www.r-project.org/ Date of citation, May 18, 2018.

[28] Berkley JA, Ngari M, Thitiri J, Mwalekwa L, Timbwa M, Hamid F, et al Daily co-trimoxazole prophylaxis to prevent mortality in children with complicated severe acute malnutrition: A multicentre, double-blind, randomised placebo-controlled trial. Lancet Glob Health. 2016; 4:e464–e473. 10.1016/S2214-109X(16)30096-1 27265353 PMC6132285

[29] Irena AH, Mwambazi M, Mulenga V. Diarrhea is a major killer of children with severe acute malnutrition admitted to inpatient set-up in Lusaka, Zambia. Nutr J. 2011; 10:110 10.1186/1475-2891-10-110 21989455 PMC3214843

[30] Talbert A, Thuo N, Karisa J, Chesaro C, Ohuma E, Ignas J, et al Diarrhoea complicating severe acute malnutrition in Kenyan children: a prospective descriptive study of risk factors and outcome. PLoS ONE. 2012; 7:e38321 10.1371/journal.pone.0038321 22675542 PMC3366921

[31] Kerpel-Fronius E, Jani L, Fekete M. Disaccharide malabsorption in different types of malnutrition. Ann Paediatr. 1966; 206: 245–257. 6012738

[32] Kukuruzovic RH, Brewster DR. Milk formulas in acute gastroenteritis and malnutrition: A randomized trial. J Paediatr Child Health. 2002; 38:571–577. 10.1046/j.1440-1754.2002.00044.x 12410869

[33] Matarese LE. Nutrition and fluid optimization for patients with short bowel syndrome. J Parenter Enter Nutr. 2013; 37:161–170. 10.1177/0148607112469818 23264168

[34] de Laffolie J, Naim HY, Rudloff S, Zimmer K-P. Starch Tolerance and the Short Bowel. J Pediatr Gastroenterol Nutr. 2018; 66 Suppl 3:S68–S71. 10.1097/MPG.0000000000001962 29762383

[35] Bartels RH, Meyer SL, Stehmann TA, Bourdon C, Bandsma RHJ, Voskuijl WP. Both Exocrine Pancreatic Insufficiency and Signs of Pancreatic Inflammation Are Prevalent in Children with Complicated Severe Acute Malnutrition: An Observational Study. J Pediatr. 2016; 174:165–170. 10.1016/j.jpeds.2016.04.013 27178623

[36] Schneider RE, Viteri FE. Luminal events of lipid absorption in protein-calorie malnourished children; relationship with nutritional recovery and diarrhea. II. Alterations in bile acid content of duodenal aspirates. Am J Clin Nutr. 1974; 27:788–796. 10.1093/ajcn/27.8.788 4211017

[37] Kimutai D, Maleche-Obimbo E, Kamenwa R, Murila F. Hypo-phosphataemia in children under five years with kwashiorkor and marasmic kwashiorkor. East Afr Med J. 2009; 86:330–336. 20499782 10.4314/eamj.v86i7.54147

[38] Spoelstra MN, Mari A, Mendel M, Senga E, van Rheenen P, van Dijk TH, et al Kwashiorkor and marasmus are both associated with impaired glucose clearance related to pancreatic beta-cell dysfunction. Metabolism. 2012; 61:1224–1230. 10.1016/j.metabol.2012.01.019 22386944

[39] Versloot CJ, Voskuijl W, van Vliet SJ, van den Heuvel M, Carter JC, Phiri A, et al Effectiveness of three commonly used transition phase diets in the inpatient management of children with severe acute malnutrition: a pilot randomized controlled trial in Malawi. BMC Pediatr. 2017; 17:112 10.1186/s12887-017-0860-6 28446221 PMC5406940

