# Supplementary material for: A reduced-carbohydrate and lactose-free formulation for stabilization among hospitalized children with severe acute malnutrition: A double-blind, randomized controlled trial
Source: PLoS Med. 2019 Feb 26;16(2):e1002747. doi: 10.1371/journal.pmed.1002747 (PMC6390989; doi:10.1371/journal.pmed.1002747)
Supplement: S1 Protocol — SAM, severe acute malnutrition. (DOCX) [file pmed.1002747.s002.docx]

**1)** **Title**

**Randomised controlled trial of a reduced carbohydrate formulation of
F75 therapeutic milk among children with severe acute malnutrition.**

## 2) Investigators

## KEMRI

## James Berkley^1^ (PI Kilifi)

Johnstone Thitiri^1^

Moses Ngari^1^

Greg Fegan^1^

Laura Mwalekwa^1^

Rehema Ali^1^

Martha Mwangome^1^

Fauzat Hamid^1^

Betty Owor^1^

**Non-KEMRI**

Victor Bandika^3^ (PI Mombasa)

Anisa Omar^4^

Patrick van Rheenen^5^

Anneke Muller Kobold^5^

Marko Kerac^6^

Wieger Voskuijl^7^ (PI Malawi)

Andre Briend^8^

Robert Bandsma^9^

Neema Mturi^1,2^

1. KEMRI CGMR-Coast, PO Box 230, Kilifi, 80108.
2. Consultant Paediatrician and Head of Department. of Paediatrics, Kilifi County Hospital
3. Consultant Paediatrician and Head of Department of Paediatrics, Coast General Provincial Hospital, Mombasa
4. Consultant Paediatrician and Kilifi County Director of Health, Kilifi County Government
5. Dept. of Gastroenterology, University Medical Center Groningen - University of Groningen
6. College of Medicine, Blantyre, Malawi
7. Head of Moyo Nutritional Rehabilitation Unit and Senior Lecturer, Department of Paediatrics and Child Health, College of Medicine, University of Malawi, Blantyre, Malawi
8. Department of Nutrition, Exercise and Sports. University of Copenhagen, Denmark
9. The Hospital for Sick Children, Toronto, Canada.

**3) Summary**

Inpatient treatment for complicated severe acute malnutrition (SAM) continues to have a high mortality in Africa. This is partly because children are commonly brought for admission because they are seriously ill, rather than being brought to hospital because of malnutrition alone. Mortality rates are especially high where SAM is complicated by HIV or TB. The early phase of inpatient nutritional treatment for severe acute malnutrition is based on a low-protein milk known as F75, which is given to improve metabolic homeostasis prior to the re-feeding to achieve catch-up growth. F75 provides a high proportion of energy from carbohydrates, including sucrose, lactose and maltodextrin. However, malabsorption of different types of carbohydrates, but lactose in particular, is known to occur in SAM and may lead to osmotic diarrhoea. Diarrhoea is common in children with SAM and is associated with increased mortality. Furthermore, switching from a catabolic state to a high energy diet that consists of predominantly carbohydrates can lead to ‘re-feeding syndrome’ that may lead to severe electrolyte abnormalities and multiple organ dysfunction.

The aim of this trial is to determine whether reducing the carbohydrate content of F75, and removing lactose, improves the stabilisation of severely malnourished children. The trial will involve randomising children who are eligible to receive F75 milk to either the current formulation or a revised formulation. Both formulations will be given according to current recommendations regarding frequency of feeding and caloric value. Since the purpose of F75 is to stabilise the child metabolically and biochemically, the primary endpoint of the trial will be time to stabilisation (the end of the first phase of treatment for severe acute malnutrition). Blood and stool samples at admission and after three days will be used to determine the effects on carbohydrate and fat malabsorption and evidence of the re-feeding syndrome. Children will be followed up until discharge from hospital. The project has been planned in consultation with the World Health Organisation (WHO) and, if the revised formulation of F75 results in improved outcomes, will lead to a global change in recommendations for its formulation.

**4) Introduction**

Admission to hospital with complicated severe acute malnutrition (SAM) in Africa commonly has a case fatality of 10-30%.[^1-3^](#_ENREF_1) Importantly, children are usually admitted to hospital because they are severely ill rather than for malnutrition alone. Mortality may be improved to some extent by adherence to WHO recommended management,[^1^](#_ENREF_1)^,^[^2^](#_ENREF_2) but the direct application of these guidelines to all contexts is controversial.[^4^](#_ENREF_4) The WHO guidelines were designed on the basis of historical data on nutrient requirements and medical treatments and almost none of the recommendations are supported by evidence from clinical trials.

For the treatment of complicated inpatient SAM, the guidelines consist of three distinct phases of treatment:[^5^](#_ENREF_5) phase 1 or stabilization phase where a low protein, liquid diet (F75) is introduced with a reduced energy intake (80-100 kcal/kg/day). Once a child has stabilised, there is a ‘transition phase’ consisting of either ready to use therapeutic foods (RUTF) with supplemental F75 or alternatively another milk formula known as F100. RUTF is a peanut based, energy dense supplement used to obtain catch-up growth. F100 is a liquid formula with a higher energy density and protein content than F75. The caloric intake is increased daily to a maximum of 130 kcal/kg/day. Finally, ‘Phase 3’ is the recovery phase during which the aim is to achieve catch-up growth with either RUTF or F100.

Typically, the highest mortality rate is found in the early phases of treatment. Children who fail treatment early often have profuse diarrhoea, signs of circulatory insufficiency which is hard to treat. The most recent reports from Zambia and Kenya note a prevalence of diarrhoea of more than 60% amongst children with SAM and it is associated with increased mortality.[^6^](#_ENREF_6)^,^[^7^](#_ENREF_7) Currently at Kilifi County Hospital and Coast Provincial General Hospital, ~20% of children are given either a diluted or lactose-free milk feed during rehabilitation of SAM (unpublished data), although there are no specific guidelines for this. Diarrhoea may be caused by a viral or bacterial gastroenteritis, sepsis, or may be nutritionally induced (osmotic). There are no routinely available tests to distinguish osmotic from infective or other causes of diarrhoea in hospitals in sub-Saharan Africa. Rotavirus vaccine will be introduced in 2014 and its contribution to diarrhoea amongst children with SAM is unknown.

The proportion of energy derived from carbohydrates in F75 is high. The carbohydrates in F75 milk (as well as F100 and RUTF) consist of a mixture of maltodextrin, sucrose and lactose. Disaccharides such as maltose, lactose or sucrose are normally hydrolysed into monosaccharides by disaccharidases localized at the tip of small intestinal villi. The monosaccharides such as glucose and galactose can then be transported across the apical membrane through Na^+^ dependent glucose transporter, whilst fructose makes use of a facilitative fructose transporter. There is limited information on the intestinal function and intestinal carbohydrate absorption in malnourished children. However, data from Jamaica and South Africa suggest that there is impaired absorption of disaccharides (lactose and sucrose), regardless of the presence or suspicion of gastroenteritis.[^8^](#_ENREF_8)^,^[^9^](#_ENREF_9) Limited histological evidence has shown intestinal atrophy in children with SAM.[^10-12^](#_ENREF_10) These data are consistent with clinical signs of lactose malabsorption found in children with severe malnutrition.[^13^](#_ENREF_13) Recently, evidence of impaired absorption of monosaccharide glucose in children with SAM was reported in Malawi.[^14^](#_ENREF_14)

Apart from diarrhoea, early deterioration may also be related to severe metabolic derangements due to a sudden change from a catabolic to an anabolic state, resulting in refeeding syndrome.[^15^](#_ENREF_15) Refeeding syndrome is characterized by hypophosphataemia, hypokalaemia, hypomagnesaemia and sodium retention. These severe electrolyte disturbances can lead to impaired cardiac, pulmonary and neurological function and are often hard to treat. By receiving energy predominantly from sugars, pancreatic insulin secretion is increased which induces uptake of electrolytes including phosphate and potassium, into cells. Furthermore, as protein synthesis is stimulated, increased production of adenosine tri-phosphate (ATP) leads to a higher cellular demand for phosphate.[^15^](#_ENREF_15) Although insulin secretion appears to be partially impaired in the early stages of refeeding, hypophosphatemia is a common feature during refeeding of malnourished children, and is associated with mortality.[^16-18^](#_ENREF_16)

In this trial, we aim to evaluate the outcome of using a revised formulation of F75 milk with reduced carbohydrate composition and without lactose, compared to the current formulation of F75 during the initial stabilisation period amongst children with severe acute malnutrition. In the new formulation, more will be provided by lipids and the total energy provided will be unchanged. The trial will be undertaken in two hospitals in Kenya and one hospital in Malawi.

**5) Justification**

There are theoretical reasons why carbohydrates in F75 might be associated with the development of diarrhoea and refeeding syndrome in severely malnourished children. However, there are no data from trials to inform the optimal carbohydrate content in therapeutic diets for malnourished children. This trial has the potential to improve treatment guidelines, and thereby the outcomes of malnourished children. Amongst children with SAM who present with, or develop diarrhoea after admission, it is unknown what proportion are due to infective and non-infective causes.

## 6) Null Hypothesis

## Reducing carbohydrates in F75 milk has no effect on the rate of stabilisation amongst children with SAM admitted to hospital.

## 7) Objectives

**General objective:**

- To optimise re-feeding diet for severely malnourished children to achieve highest recovery rate and lowest rate of complications.

**Specific objectives:**

- To determine the efficacy on time to stabilisation of a formulation of F75 milk with reduced carbohydrate content compared to the current standard formulation of F75 amongst hospitalised children with SAM.
- To determine the frequency of the biochemical abnormalities associated with re-feeding syndrome, osmotic and infective diarrhoea amongst hospitalised children with SAM.

**8) Design**

The trial is designed as a multicentre randomized double-blind intervention trial. The trial will examine the time to reach the transition phase amongst children with severe acute malnutrition receiving F75 milk with the current standard carbohydrate content and those receiving a modified formulation of F75 with reduced carbohydrate content without any lactose. The total energy, protein and micronutrient content of the two formulations will be the same. All other aspects of care will be the same in both arms, and be provided according to the Kenyan National Guidelines for the Integrated Management of Acute Malnutrition.[^20^](#_ENREF_20)

***a) Study Sites***

### Paediatric wards of Kilifi County Hospital and Coast Provincial General Hospital, Kenya and Queen Elizabeth Hospital, Blantyre, Malawi.

### ***b) Study population***

***Inclusion Criteria:***

- Age 6 months to 13 years.
- Severe malnutrition defined as: mid upper arm circumference (MUAC) <11.5cm if less than 5 years old;[^19^](#_ENREF_19) or weight for height Z score <-3; or kwashiorkor as defined in the current Kenyan and WHO guidelines.[^20^](#_ENREF_20)
- Admitted to hospital because of medical complications as defined in the current WHO guidelines or fails an appetite test if there are no complications.[^20^](#_ENREF_20)
- Eligible to start F75 milk by current Kenyan and WHO guidelines.[^20^](#_ENREF_20)

***Exclusion Criteria:***

- Declined to give informed consent
- Known allergy to milk products
- Any other reason the consenting investigator thinks that in the child’s best interests it inappropriate for them to take part.

***c) Sampling***

Children admitted with severe malnutrition, defined by anthropometry according to WHO guidelines, will be recruited at CPGH, Mombasa, Kilifi County Hospital and Queen Elizabeth Central Hospital (QECH), Blantyre, Malawi.

*Sample size calculation*

Based on clinical data from the actual sites, we have observed that around 50% of patients to have transitioned to the second phase of treatment by day 5. We aim for a 10% absolute increase in percentage transitioned at day 5 to 60%, which gives a hazard ratio of 1.35. Sample size for survival time was calculated using the *stpower* command in Stata version 12. Using α=0.05, 80% power, the number of patients required to demonstrate a hazard ratio of 1.35 is 381 per arm. We will aim at 420 per arm to allow for contingencies. It is anticipated that 400 children will be enrolled at CPGH, 120 at KCH and 320 at QECH. However, these figures will be adjusted in line with actual recruitment.

***d) Procedures***

*Sponsor, steering and monitoring committees*

The trial sponsor will be the University of Oxford. The sponsor will be responsible for ensuring appropriate arrangements are in place for the initiation, management and financing of the trial, the trial is conducted to a high scientific quality and the rights of participants are protected. A trial steering committee (TSC) will be appointed to oversee the running of the trial. A data safety and monitoring committee (DSMC) will be convened. The focus of the DSMC is safety, the overall integrity of the study, and its continued relevance and ability to answer the primary objective. The TSC will be advised on the study progress and continuation by the DSMC. One interim unblinded analysis will be conducted by the DSMC during the trial after 50% of participants are recruited. A recommendation to the sponsor to discontinue recruitment, in all patients or in selected subgroups, may be made by the TSC on advice from the DSMC if the data provide proof beyond reasonable doubt that one of the treatment arms is better in terms of the primary outcome or safety guided by the Haybittle-Peto criteria. A local safety monitor (LSM) will be appointed to provide independent consultation on clinical care, management of clinical cases. The LSM will also retain a sealed code break envelope and in may be able to un-blind specific individual allocation if required for safety considerations.

*Training and setting up*

At each site an initial period will be spent training staff in anthropometry and management of severe malnutrition in order that children will be managed in line with WHO recommendations to the best standard available at each hospital. This will be followed by study-specific training. Staff at all sites will be trained on the background to the trial; scientific and ethical aspects of clinical research and trials; ICH GCP; anthropometry; data collection; communication and consenting procedures.

*Screening*

The screening process will be based on the severe malnutrition criteria which is part of routine hospital admission procedure for all children, as specified in national guidelines for care of severe malnutrition.

*Enrolment*

Enrolment will occur at the time that F75 milk would normally be started. Details of the study will be communicated to the parents/guardians of potential participants. Prior to seeking informed consent, information on benefits and risks will be provided to parents or carers in their local language and written informed consent sought. In order to ensure patients receive optimal care, to avoid delaying urgent feeding (i.e. as per WHO recommendations to prevent hypoglycaemia), assent for participation will be sought, and then written consent requested after the initial stabilisation. In the rare event that a child for whom assent for participation has been given, but who dies before written consent has been given, data and samples will be used for the study but parents and caregivers will not be asked for written consent at this time of grief. This approach is being successfully used in another study SSC#1778.

Baseline data of prognostic importance will be collected, including vaccination status. A blood sample for haemoglobin and storage of plasma, will be collected at enrolment (up to 5ml, not exceeding 1ml/kg body weight).

*Investigational product*

We will evaluate the effect of a lactose free, reduced carbohydrate F75 compared to standard F75:

1. F75 with 63% of total energy from carbohydrates, including 10% of energy from lactose (standard F75).

2. F75 with 43% of total energy from carbohydrates, without any lactose, and providing the same amount of energy as standard F75 by increased lipid in the form of medium chain triglycerides.

Although increasing energy from lipids to 52% could potentially result in some fat malabsorption leading to steatorrhoea, it will not cause osmotic diarrhoea. Steatorrhoea does not cause severe dehydration and electrolyte imbalances. In the standard RUTF that currently used in severely malnourished children, the energy content provided by lipids is ~60%, which is well tolerated in this patient group.

Standard F75 and reduced carbohydrate F75 will be manufactured to GMP standards by Nutriset®, France who are the current supplier to UNICEF and the Kenyan and Malawian national programmes, and through these channels, to the study hospitals. Protein and micronutrient components will be unchanged and taste is very similar. The study products will be identically packaged, marked ‘for research use only’. Dosing will be as per Kenyan and Malawian recommendations: 130 kcal/kg/day per day, divided into 8 feeds per 24 hours.

|  | % Energy | | Quantity: g/1000ml | |
| --- | --- | --- | --- | --- |
|  | **Standard F75** | **Modified F75** | **Standard F75** | **Modified F75** |
| Protein | 5.3% | 5.3% | 9.9 | 9.9 |
| Lipid | 31.5% | 51.7% | 26.3 | 43.1 |
| Carbohydrate | 63.2% | 43.0% | 118.5 | 80.6 |
| **Total** | **100%** | **100%** | **154.7** | **133.6** |
|  |  |  |  |  |
| Lactose | 9.9% | 0% | 18.6 | 0 |
| Sucrose | 3.6% | 3.6% | 6.8 | 6.8 |
| Maltodextrin | 50% | 39% | 93.2 | 73.9 |

*Formulations of standard F75 and modified F75*

*Randomisation*

Sequential study numbers will be generated for each site according to a prepared blocked randomisation list of random block sizes before the study begins. Randomisation codes linking allocation to study number will be held by the local safety monitor and chair of the DSMC be opened only in case an individual safety concern or until the database is unlocked. Children will be allocated study numbers sequentially within each site, thus randomly allocating the intervention and maintaining blinding. Group allocation will remain unknown to participants and trial personnel, including the trial-coordinator and principal investigator. Code breaks will be provided to the research team, to be used only in the case of an emergency; 24 hour access will be provided to these code breaks by mobile phone.

*Clinical care*

Children will be managed to the best standard available in each facility, in line with the Kenyan and WHO recommendations. HIV testing by rapid antibody test is routinely offered by the hospitals involved in the trial at admission to all severely malnourished children through ‘provider initiated testing’ according to the current National Guidelines for HIV Testing and Counselling in Kenya (2008) and appropriate follow up tests and referral for care are undertaken according to the results.

*Follow up*

Children will be examined daily whilst in hospital and clinical status recorded on a standardised proforma (appendix d). A blood sample and stool sample will be taken at day 3 to assess for metabolic effects of refeeding and carbohydrate malabsorption. If children deteriorate during the transition phase or phase 2 and are placed back on phase 1, they will receive the same F75 intervention diet as allocated during the initial treatment. The decision to discharge will be made by the usual clinical team rather than trial personnel. Food supplements and/or nutritional counselling and follow up in the nutritional clinic that are the current standard of care at each institution will also be done at discharge. No study follow up after discharge will be done.


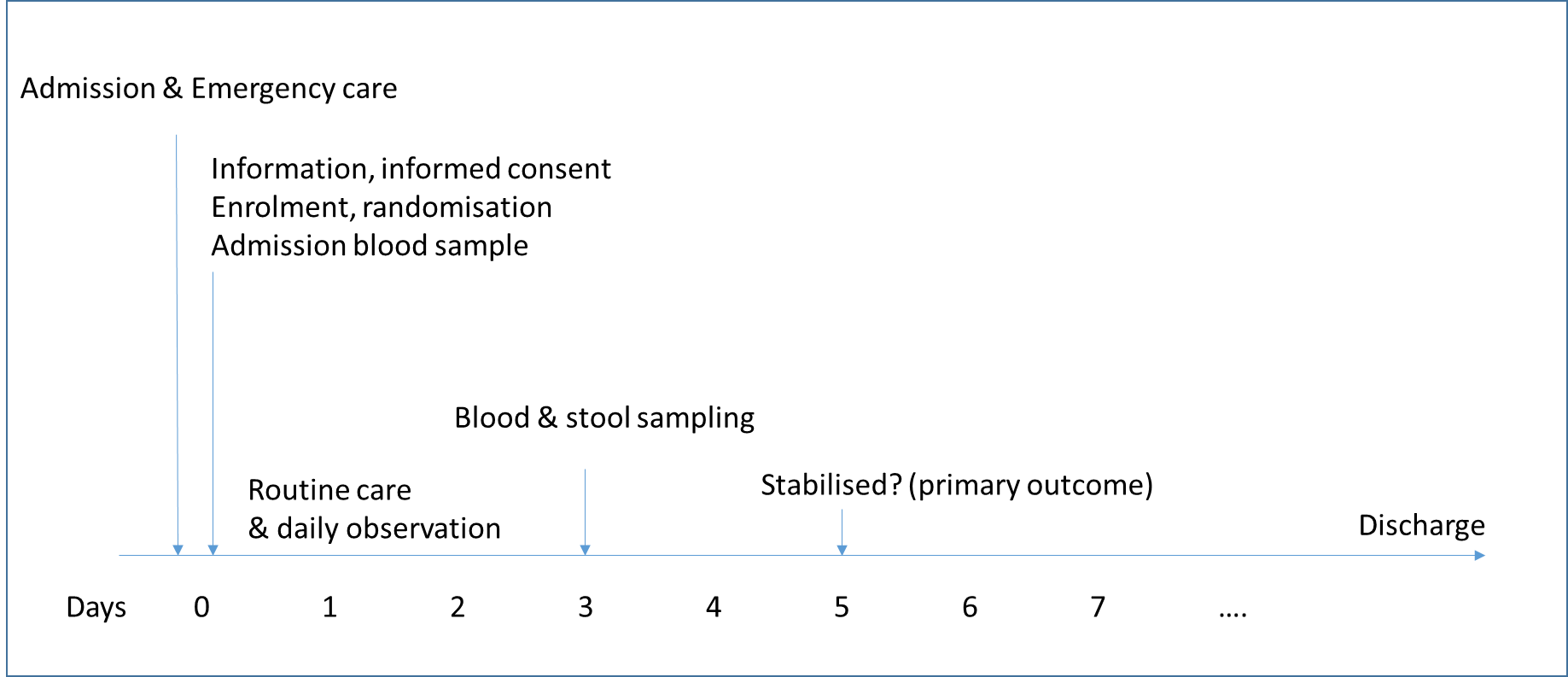


*Outcome measures*

*Primary outcome:* the choice of primary outcome is based on the therapeutic purpose of F75, which is to achieve stabilisation and thereby allow a child to progress to feeds with higher protein and energy content to enable catch-up growth.

The primary outcome is stabilisation, defined as having reached the second (transition) phase of treatment. The criteria for stabilisation will be according to WHO guidelines:

Absence of any WHO danger or emergency signs: obstructed breathing, respiratory distress, cyanosis, shock (delayed capillary refill plus fast & weak pulse plus temperature gradient), severe anaemia (Hb<5g/dl), congestive cardiac failure, impaired consciousness, convulsions, severe dehydration, profuse watery diarrhoea, vomits everything, hypothermia.

*and*

If there is oedema at baseline, loss of oedema defined as improving from a severe +++ oedema (severe: generalized bilateral pitting oedema including feet, legs, arms and face) to ++ oedema (moderate: no upper arm or upper leg oedema and no facial oedema or from ++ oedema to + (mild: only feet/ankle oedema) or none;

*and*

Tolerating full prescribed volume of F75 feeds and observed to be completing the feeds.

For analysis, children who die before stabilisation will be classified as ‘not transitioned’.

*Pre-specified secondary outcomes*:

1. Number days with diarrhoea (3 or more loose stools within 24h);
2. Number days requiring rehydration fluids (ReSoMal or IV fluids);
3. Percentage change in weight between admission and day 5;
4. Changes in sodium, potassium, magnesium, calcium, phosphate and albumin between admission and day 3;
5. Faecal electrolytes, pH and fat at day 3;
6. numbers of episodes of ***new onset*** severe clinical deterioration accompanied by one or more of the following features: shock (fast and weak pulse and limb versus core temperature gradient and capillary refill time>3 seconds), respiratory distress (subcostal chest wall indrawing, hypoxaemia (SaO_2_) or requiring oxygen); impaired consciousness (Blantyre coma score<4) or hypoglycaemia (<3.0 mmol/l);
7. mortality until discharge;
8. time to discharge from hospital in days;
9. total days spent in the stabilization phase, including periods when the child may go back to the stabilization phase during a deterioration;
10. the proportion children with a diarrhoeal pathogen detected, (see laboratory procedures on page 8).
11. Exploratory analysis of plasma and faecal markers of gut inflammation and permeability (see laboratory procedures on page 8).
12. Gut microbiome (see laboratory procedures on page 8).
13. Metabolite profiles in plasma and stool (see laboratory procedures on page 8).

***Monitoring of adverse events***

This group of children are expected to have a high frequency of adverse events regardless of this intervention. There is no specific toxicity profile associated with F75 milk. The study team will monitor children through daily clinical examination. Laboratory tests including a full blood count, electrolytes, blood glucose and liver function tests will be done as guided by requirements for clinical care. Serious adverse events, include events that are life-threatening or prolong hospital admission are pre-specified as secondary endpoints number vi) and vii) above. These will be recorded and entered on the trial database. A summary of serious adverse events will be reported quarterly, and examined in an un-blinded analysis by the DSMB at the trial mid-point and again at the end of the trial.

***Monitoring of study procedures***

Study monitoring will be conducted to ensure that the conduct of the study complies with ICH Guidelines and the trial’s standard operating procedures. In Kenya, the trial will be monitored by the KEMRI/Wellcome Clinical Trials Facility Monitoring Team. A comprehensive study initiation visit will be done at which all staff will be trained in protocol specific procedure, and check that all trial logistics are in place. Routine monitoring will be conducted every three months or more frequently if required. The monitor’s role is part of the quality system that will ensure that the study file is maintained with updated master file documents, that all participants have dully completed informed consent and that staff on the study are following their SOP’s in accordance to protocol and applicable regulatory norms.

### ***Laboratory procedures***

Blood tests for clinical care such as full blood counts, electrolytes and liver function will be undertaken as usually done for clinical care at each site. In addition, a plasma sample will be stored at admission and day 3 in order to measure sodium, potassium, calcium, magnesium, phosphate and albumin by standard methods at the end of the study in Kilifi. Plasma samples from Kenya and Malawi will be analysed for evidence of gut dysfunction and permeability including IL-1b, IL-6, IL-8, IL-10, TNFα, IFNϒ, LPS, sCD14 and intestinal fatty acid binding protein by ELISA and multiplex bead array methods in Kilifi and/or at the Hospital for Sick Children, Toronto, Canada.

Stools will be homogenised by stirring and stored at minus 80 degrees C in sterile tubes in Kilifi. At the end of the trial, stored stools from admission and day 3 will be analysed. Stools will be analysed by multiplex PCR for diarrhoeal viral pathogens in Kilifi using our existing long-term surveillance platforms and by light microscopy following filtration and concentration processing for helminths and protozoa. These assays will be performed in Kilifi. Analyses will further consist of specialised tests: faecal electrolytes as a marker of osmotic vs. secretory diarrhoea, faecal fat, faecal pH as markers of carbohydrate malabsorption and calprotectin as a marker of intestinal inflammation and will be performed together with samples from Malawi in Kilifi and at the University of Groningen, the Netherlands. Analyses of the faecal bacterial microbiome using pyrosequencing and biomarkers of intestinal (dys)-function using mass spectrometry will be analysed in Kilifi and/or at the Hospital for Sick Children, Toronto, Canada. Since these techniques are not yet fully established in Kilifi, skills and technology will be transferred from Canada to the Netherlands to Kilifi, in order that these assays will be done locally with validation from the eternal laboratories. Remaining samples will be stored in Kilifi for up to 5 years future studies of pathophysiology, subject to approval by SSC and ERC.

## 9) Data

### (a) Data storage

The principal and site investigators will maintain appropriate medical and research records for this trial, in compliance with ICH E6 GCP and regulatory and institutional requirements for the protection of confidentiality of subjects. Permission may be granted for the examination, analysis, verification and reproduction of any records and reports that are important to evaluation of a clinical trial. Any party (e.g., domestic and foreign regulatory authorities, sponsor's monitors and auditors) with direct access should take all reasonable precautions within the constraints of the applicable regulatory requirement(s) to maintain the confidentiality of subjects' identities and sponsor’s proprietary information. All records will be kept in a locked filing cabinet, which is accessed only by the investigators and the study nurses. All computer entry and networking programs will be done with coded numbers and initials only.

### (b) Data management/analysis

The trial will use the GCP-compliant Openclinica (<https://openclinica.com/> ) database system, which will be hosted at KWTRP in Kenya. Originally developed for small oncology trials, this system has become the leading open-source clinical data capture system for clinical trials. It allows secure remote data entry online from the sites, provides a real-time reporting system and flagging of inconsistencies and database changes for monitoring. We have used this system for several large clinical trials, which have been programed and maintained by the data manager who will be working on this trial. The trial will be register on clinicaltrials.gov along with pre-specified endpoints. Data cleaning and record verification will be completed and a written analysis plan agreed before the database is un-blinded.

Descriptive statistics on baseline characteristics of the participants in each arm will be provided. Bias will be determined by maintaining a screening log of all potentially eligible participants and those who were excluded or randomized into the two arms. This will be presented as a flow diagram in the final published report according to CONSORT statement guidelines.

The primary outcome will be assessed using a Kaplan-Meier analysis and Cox proportional hazards analysis comparing the rate of stabilisation between arms. Differences between arms in the number of days to stabilisation will be compared using a two tailed Mann-Whitney *U* test. Children who die prior to stabilisation will be included in the primary analysis as ‘not stabilised’. Secondary endpoints with binary outcomes (e.g. overall mortality) will be analysed using contingency tables and a chi squared test. Where adjustment for baseline parameters is required, relative risks will be calculated. For continuous variables and count data (e.g. days receiving rehydration fluids or days with diarrhoea), a suitable general linear model (e.g. linear regression, Poisson regression) will be used. For time to discharge, Kaplan-Meier and Cox proportional hazards analysis will be used. These analyses are conducted on the intention-to-treat principle and are considered the confirmatory analysis. We will conduct tests of heterogeneity of effect, and effect modification for both kwashiorkor and HIV infection, which are considered, *a priori,* to potentially alter the response to F75.

**10) Time Frame**

|  | 2014 | | | | 2015 | | | |
| --- | --- | --- | --- | --- | --- | --- | --- | --- |
|  | Q1 | Q2 | Q3 | Q4 | Q1 | Q2 | Q3 | Q4 |
| Approvals |  |  |  |  |  |  |  |  |
| Training & Setup |  |  |  |  |  |  |  |  |
| Recruitment & Follow up |  |  |  |  |  |  |  |  |
| Laboratory work |  |  |  |  |  |  |  |  |
| Analysis/reporting/dissemination |  |  |  |  |  |  |  |  |

**11) Ethical Considerations**

### (a) Human subjects

*i.* “*First do no harm.”*

*ii. Risks and benefits*

Modified F75 milk is likely to be a low risk intervention. However, the patient population has a high background risk of morbidity and mortality, and is economically and socially disadvantaged. The risks of the proposed blood sampling are low.

The bodies involved in approval or oversight of the study are: The KEMRI Scientific Coordinating Committee (SCC), Centre for Geographic Medicine Research (Coast), Kilifi; the KEMRI Scientific Steering Committee (SSC), Nairobi, the KEMRI Ethical Review Committee (ERC); the Oxford Tropical Research Ethics Committee (OXTREC); the Trial Steering Committee (TSC); the Data Safety Monitoring Committee (DSMC); and the Clinical Trials Facility monitoring team, CGMRC.

Children will benefit from the additional clinical staff, who will also assist in the care of non-trial participants who are severely malnourished. Children in the trial will benefit from close monitoring of their progress. The benefit to the community will be in developing improved care for malnourished children.

Community engagement will be focussed on the hospital setting since this is an entirely hospital-based study. It will be through established regular meetings with the hospitals involved, specifically through staff in the wards and nutrition clinics. Engagement with the wider community will be through the activities of the programme’s Community Liaison Group, which includes regular meetings with KEMRI-Community Representatives. Through these activities, information and feedback from the community. Currently, information on the research activities of KEMRI is given to the community through the activities of the programme’s Community Liaison Group, including regular meetings with the KEMRI-Community Representatives, and meeting with the DHMTs, hospital and peripheral health staff and local administrative leaders.

iii. *Informed consent*

The parent or guardian will receive an explanation of the trial at admission and be given a chance to ask questions before written permission for their child to be included in the trial is sought by a member of the study team. It is recognised that in some cases, obtaining informed consent at admission for an acutely ill child may present challenges. Previous work on obtaining consent suggests a staged process.[^21^](#_ENREF_21) The validity of this has been assessed for use in cord blood collection in both Kilifi and Mombasa. (SSC Nos. 1215, 1778 & 1355). In situations where urgent feeding is needed, but it would be delayed by taking full informed consent, it is proposed to use a two phase consent process, where verbal assent for enrolment is taken by a clinician (appendix B) and later written consent (appendix C) is sought. If consent is refused at the second stage, any stored samples would be discarded and the child would receive standard care.

Field workers and clinical staff will be trained in administering the two stage consent procedure, following a standard operating procedure. Written consent will be taken by trained fieldworkers, recruited from the local population and fluent in the predominant local languages.

*iv. Confidentiality*

All records will be kept in a locked filing cabinet, which is accessed only by the investigators and the study nurse(s). No names will be recorded on the trial database. Every child will be allocated a unique identifying number which will be used as the linking identifier for databases. Only the investigators, the clinical monitor, the ethics committees or any other regulatory agencies will have access to the records. Every effort will be taken to maintain confidentiality. No information concerning the study or the data will be released to any unauthorized third party, without prior written approval of the sponsor.

### *v*. *New Drugs:* None, the intervention comprises a revised formulation of milk feeds.

(b) Animal subjects: Not applicable

**12) Expected application of the results**

The trial aims to directly influence policy for the treatment of severe malnutrition in children. The trial has been designed with input and endorsement from the Department of Maternal, Newborn, Child and Adolescent Health at WHO in order that it has the potential to subsequently influence policy (appendix F). Feedback to the hospitals and other stakeholders will be given, and result presented to the sites and to national and international agencies at the end of the trial.

**13) References**

**1.** Ashworth A, Chopra M, McCoy D, et al. WHO guidelines for management of severe malnutrition in rural South African hospitals: effect on case fatality and the influence of operational factors. *Lancet.* Apr 3 2004;363(9415):1110-1115.

**2.** Deen JL, Funk M, Guevara VC, et al. Implementation of WHO guidelines on management of severe malnutrition in hospitals in Africa. *Bull World Health Organ.* 2003;81(4):237-243.

**3.** Maitland K, Berkley JA, Shebbe M, Peshu N, English M, Newton CR. Children with severe malnutrition: can those at highest risk of death be identified with the WHO protocol? *PLoS Med.* Dec 2006;3(12):e500.

**4.** Heikens GT. How can we improve the care of severely malnourished children in Africa? *PLoS Med.* Feb 2007;4(2):e45.

**5.** WHO. *Pocket Book of Hospital Care for Children, 2nd edition.* Geneva2013.

**6.** Talbert A, Thuo N, Karisa J, et al. Diarrhoea complicating severe acute malnutrition in Kenyan children: a prospective descriptive study of risk factors and outcome. *PLoS one.* 2012;7(6):e38321.

**7.** Irena AH, Mwambazi M, Mulenga V. Diarrhea is a major killer of children with severe acute malnutrition admitted to inpatient set-up in Lusaka, Zambia. *Nutr J.* 2011;10:110.

**8.** James WP. Jejunal disaccharidase activities in children with marasmus and with kwashiorkor. Response to treatment. *Arch Dis Child.* Apr 1971;46(246):218-220.

**9.** Bowie MD, Barbezat GO, Hansen JD. Carbohydrate absorption in malnourished children. *Am J Clin Nutr.* Feb 1967;20(2):89-97.

**10.** Stanfield JP, Hutt MS, Tunnicliffe R. Intestinal biopsy in kwashiorkor. *Lancet.* Sep 11 1965;2(7411):519-523.

**11.** Schneider RE, Viteri FE. Morphological aspects of the duodenojejunal mucosa in protein--calorie malnourished children and during recovery. *Am J Clin Nutr.* Oct 1972;25(10):1092-1102.

**12.** Sullivan PB, Marsh MN, Mirakian R, Hill SM, Milla PJ, Neale G. Chronic diarrhea and malnutrition--histology of the small intestinal lesion. *J Pediatr Gastroenterol Nutr.* Feb 1991;12(2):195-203.

**13.** Prinsloo JG, Wittmann W, Pretorius PJ, Kruger H, Fellingham SA. Effect of different sugars on diarrhoea of acute kwashiorkor. *Arch Dis Child.* Oct 1969;44(237):593-599.

**14.** Bandsma RH, Spoelstra MN, Mari A, et al. Impaired glucose absorption in children with severe malnutrition. *J Pediatr.* Feb 2011;158(2):282-287 e281.

**15.** Kraft MD, Btaiche IF, Sacks GS. Review of the refeeding syndrome. *Nutrition in clinical practice : official publication of the American Society for Parenteral and Enteral Nutrition.* Dec 2005;20(6):625-633.

**16.** Kimutai D, Maleche-Obimbo E, Kamenwa R, Murila F. Hypo-phosphataemia in children under five years with kwashiorkor and marasmic kwashiorkor. *East Afr Med J.* Jul 2009;86(7):330-336.

**17.** Manary MJ, Hart CA, Whyte MP. Severe hypophosphatemia in children with kwashiorkor is associated with increased mortality. *J Pediatr.* Dec 1998;133(6):789-791.

**18.** Yoshimatsu S, Hossain MI, Islam MM, et al. Hypophosphatemia among severely malnourished children with sepsis in Bangladesh. *Pediatrics international : official journal of the Japan Pediatric Society.* Feb 2013;55(1):79-84.

**19.** Myatt M, Khara T, Collins S. A review of methods to detect cases of severely malnourished children in the community for their admission into community-based therapeutic care programs. *Food and nutrition bulletin.* Sep 2006;27(3 Suppl):S7-23.

**20.** WHO. Pocket book for hospital care of children: guidelines for the management of common illness with limited resources. 2005.

**21.** Vawter D, Rogers-Chrysler G, Clay M, Pittelko L, Therkelsen D, Kim D, McCullough J. A phased consent policy for cord blood transfusion. *Transfusion.* 2002;42:1268 - 1274.

**14) Budget (Kenya only)**

|  | KSh | US$ |
| --- | --- | --- |
| **Personnel** |  |  |
| Clinical & Trials staff | 9,500,000 | 115,853 |
| **Administration & Travel** |  |  |
| Stationary & communications | 250,000 | 3,050 |
| Trial documentation | 200,000 | 2,440 |
| Training | 360,000 | 4,400 |
| Engagement & Dissemination | 200,000 | 2,440 |
| Flights to Malawi | 420,000 | 5,120 |
|  |  |  |
| **Consumables/Equipment** |  |  |
| Sample collection | 270,000 | 3,600 |
| Laboratory investigations (e.g. Hb, U&E, LFT) | 800,000 | 9,750 |
| Anthropometric equipment | 78,000 | 950 |
| Computer equipment | 200,000 | 2,440 |
| Clinical care, drugs, consumables | 1,800,000 | 21,950 |
| F75 Milk | Donated by Nutriset | Donated by Nutriset |
|  |  |  |
| Subtotal | 14,078,000 | 171,853 |
| Contingency | 2,111,700 | 25,799 |
| **Total** | **16,189,700** | **197,652** |
| Funding for the project has been awarded to Dr Berkley by the Thrasher Research Foundation, USA. Other costs will be met by the core budget of the KEMRI/Wellcome Trust research Programme. | | |

**15) Justification of the budget**

Personnel: a clinical officer is needed at CPGH to provide clinical care and patient enrolment. Fieldworkers will be needed for informed consent procedures, anthropometric measurements and questionnaire administration. A hospital nutritionist and nurse on locum duty will assist with clinical care and supervise anthropometry. A data manager will design, maintain, verify, backup and secure databases and supervise data entry. A community liaison officer will provide communication with the study community and health service providers. The latter two posts will be shared with other projects.

Supplies and administration: there is a need for equipment in outpatients to carry out anthropometric measurements: weighing scales, height boards and MUAC strips. Costs of communications and trial documentation are based on previous trials. Costs for routine lab investigations to provide care, and research investigations are included. Travel costs for meetings in Malawi and with the DSMC and TSC are included.

## 16) Appendices

a) Role of each investigator

b) Assent form for trial participation

c) Informed consent information sheet and forms

d) Case record and data collection forms

## Appendix A: Role of each investigator

| **Name** | **Study Design** | **Recruitment & Data Collection Kenya** | **Recruitment & Data Collection**  **Malawi** | **Patient care Kenya** | **Patient Care Malawi** | **Trial management & Training** | **Data management** | **Oversight & Liaison** | **Laboratory analysis** | **Data analysis** |
| --- | --- | --- | --- | --- | --- | --- | --- | --- | --- | --- |
| **James  Berkley** |  |  |  |  |  |  |  |  |  |  |
| **Johnstone Thitiri** |  |  |  |  |  |  |  |  |  |  |
| **Moses  Ngari** |  |  |  |  |  |  |  |  |  |  |
| **Greg  Fegan** |  |  |  |  |  |  |  |  |  |  |
| **Laura Mwalekwa** |  |  |  |  |  |  |  |  |  |  |
| **Rehema  Ali** |  |  |  |  |  |  |  |  |  |  |
| **Martha Mwangome** |  |  |  |  |  |  |  |  |  |  |
| **Fauzat  Hamid** |  |  |  |  |  |  |  |  |  |  |
| **Betty  Owor** |  |  |  |  |  |  |  |  |  |  |
| **Neema  Mturi** |  |  |  |  |  |  |  |  |  |  |
| **Victor  Bandika** |  |  |  |  |  |  |  |  |  |  |
| **Anisa  Omar** |  |  |  |  |  |  |  |  |  |  |
| **Patrick van Rheenen** |  |  |  |  |  |  |  |  |  |  |
| **Marko  Kerac** |  |  |  |  |  |  |  |  |  |  |
| **Wieger  Voskuijl** |  |  |  |  |  |  |  |  |  |  |
| **Andre  Briend** |  |  |  |  |  |  |  |  |  |  |
| **Anneke Muller Kobold** |  |  |  |  |  |  |  |  |  |  |
| **Robert Bandsma** |  |  |  |  |  |  |  |  |  |  |

Appendix B: **Assent form for trial participation**

**F75 Milk Trial**

**For carers of children requiring urgent feeding, a brief discussion with the child or the child’s mother or carer by the admitting trial doctor or nurse must include the following phrases:**

• KEMRI works with the MoH to make sure that each child is treated to the best of our ability.

• KEMRI also conducts research on this ward to find better ways of treating children.

• This trial involves testing a new formulation of the milk called F75 that is normally given in the early phases of treatment for children with malnutrition in hospital. For children who are included, half will get the standard F75 and half will get the new formula F75 using a system based on chance (random). This doesn’t affect any other aspect your treatment in any way.

•If you agree for your child to participate, blood sample amounting to 5ml (one teaspoon) and a stool sample for research will be collected along with the routine blood tests on admission. After 3 days, a further blood sample and a stool sample will be collected.

• This research has been approved in advance by national scientific and independent ethical committees in Nairobi and a scientific committee in Kilifi.

• If you do not wish your child to participate in this research, your treatment will not be affected. If you agree, we will explain more about the research, and you are free to change your mind about participating at any time.

| Parent/carer assents to research? | Yes / No (please clearly circle Yes or No) |
| --- | --- |

"I was present when _____________________________________ was read this form and said that he or she agreed, or assented, for their child to take part in this study”.

| Signature of person who obtained assent | Print name of person who obtained assent | Date | | | | | | | | |
| --- | --- | --- | --- | --- | --- | --- | --- | --- | --- | --- |
|  |  | D | D | M | M | M | 2 | 0 | Y | Y |

If you would like to communicate with someone not involved in this study please contact**:** The Community Liaison Office hotline number 0723 342 870 between 8am and 5pm. Or The Secretary, KEMRI Ethics Committee, P.O Box 54840-00200. Nairobi. Tel no: 020 272 2541. Mobile: 0722205901 or 0733400003.

Appendix C: **Participant information and informed consent forms**

**F75 Milk Trial**

| Investigator | Institutional Affiliation |
| --- | --- |
| James Berkley, Johnstone Thitiri^,^ Moses Ngari, Greg Fegan,^,^Laura Mwalekwa ,Rehema Ali^,^ Martha Mwangome^,^ Fauzat Hamid, Betty Owor, Neema Mturi | KEMRI/Wellcome Trust Research Programme, Kilifi. |
| Victor Bandika Anisa Omar  Patrick van Rheenen  Anneke Muller Kobold  Marko Kerac  Wieger Voskuijl  Andre Briend  Robert Bandsma | Coast General Provincial Hospital, Mombasa  Kilifi County Director of Health  University Medical Center Groningen – Holland  University Medical Center Groningen - Holland  College of Medicine, Blantyre, Malawi  Queen Elizabeth Central Hospital, Malawi  University of Copenhagen, Denmark  The Hospital for Sick Children, Toronto, Canada. |

What is KEMRI?

KEMRI is part of the Ministry of Health that carries out research with the aim of finding better ways of prevention and treating illness in the future, for everybody’s benefit. We are asking your permission for your child to participate in a study.

What is this research about?

Your child has been admitted to this hospital and found to have malnutrition. Malnutrition is initially treated with special milk called F75. This milk is designed to give your child the nutrients that s/he requires to begin to recover from malnutrition. We want to try a different recipe for the F75 milk to see if it can help children recover more quickly. In the new recipe a sugar called lactose has been reduced. Some children are already treated with ‘lactose-free’ milks. We would like to see if more children should be receiving it. In order to find out whether the new recipe is effective, half the children in this study will receive the new recipe and half will receive the current recipe. The decision on which child will get the new recipe will be by chance. The recipes look similar and neither you nor any of our study staff will know which recipe your child has received until the end of the study.

What will it involve for me/my child?

1. Your child will continue to receive usual care and treatment for the condition with which he/she is admitted until discharge. We will document the daily progress of your child.
2. Your child will be prescribed one of the F75 milks. It is important not to share the milk with other children or use another child’s milk.
3. We will take small blood samples (5ml, a teaspoon) from your child now, together with the routine blood tests, and again after 3 days. We will also collect a stool sample at admission and after 3 days.
4. We do not need to follow you child up after discharge. Once discharged, your child will have completed the study.

Are there any risks from my child’s participation?

The new recipe of F75 milk is not expected to cause any problems. Drawing of blood from your child will cause some discomfort and we will do this at the time of routine sampling where possible. We will make sure that your child receives proper care for malnutrition and we will document and act on any problems noted with your child during the course of treatment and participation in the study.

Are there any benefits for my/my child’s participation into this study?

The benefits for your child taking part are that your child will be closely monitored during the trial. The results of this study will be beneficial to society at large as they may influence how we care for children with malnutrition.

What will happen if I do not agree to participate?

Participation in research is voluntary. You are free to decide if you want your child to take part in research or not. Your child will still receive the recommended standard care whether or not you agree to take part. If you have decided to participate, you can change your mind any time and withdraw your child from the study. This will not affect your child’s care now or in the future.

What will happen to the blood and other samples?

During this research a small volume of blood and stool may be stored for testing how the body is responding to the milk and if infections are causing diarrhoea. Some of these tests will be done overseas at the Hospital for Sick Children, Toronto, Canada or at the University Medical Centre Groningen, Holland if you permit us to do that. Samples may be stored for up to 5 years.

Who will be involved in accessing my child’s information in this study?

All information on participants collected in this study will be stored in a confidential manner in locked, secured cabinets and password protected computers and will only be accessible to authorised study personnel. The clinical monitor, the ethics committees or any other regulatory agencies will have access to the records. Every effort will be taken to maintain confidentiality. Data will be stored to the end of the study and analysis. Any report or publications on this study will not use participant’s names or identities.

Who has authorized this research to take place?

This study has been approved by the KEMRI Ethics Review Committee in Nairobi and the Ethics Review Committees that oversee our partners in this project in Blantyre College of Medicine, Malawi; the Hospital for Sick Children Toronto, Canada and The University of Oxford, UK. Oversight of the study in Kenya is provided by the KEMRI Ethics Review Committee.

What if I have any questions?

You can ask any of our workers anytime. You can also communicate with those involved in taking care of your child and this study, or Dr James Berkley KEMRI-Wellcome Trust, PO Box 230, Kilifi Kenya Tel no. 041 7522 063, Johnstone Thitiri KEMRI-Wellcome Trust, PO Box 230, Kilifi Kenya Tel. no. 041 7522 063. You may also reach the study team on add mobile number

If you would like to communicate with someone not involved in this study please contact**:** The Community Liaison Office hotline number 0723 342 870 between 8am and 5pm or The Secretary, KEMRI Ethics Committee, P.O Box 54840-00200. Nairobi. Tel no: 020 272 2541. Mobile: 0722205901 or 0733400003. The KEMRI ERC may also be contacted by email at: [ercadmin@kemri.org](mailto:ercadmin@kemri.org)

**KEMRI-Wellcome Programme Consent Form: F75 Milk Trial**

I, being the parent/ guardian of ______________________________________ (name of the child), have been given the explanation about this study.

I have understood what has been explained to me and my questions have been answered satisfactorily. I understand that I can change my mind any time and it will not affect the management of my child.

Please fill the boxes as required:

Please tick. I have agreed to allow my child to participate

Please tick. I have agreed for blood and stool samples to be stored

Please tick. I have agreed for blood and stool samples to be exported.

Parent/guardian’s signature------------------------------------------------------- Date------------------------

Parent/guardian’s name ----------------------------------------------------------- Time-------------------------

(Please print your name)

I certify that I have followed all the study procedures in the S.O.P for obtaining consent.

Coordinator/researcher’s signature--------------------------------------------- Date----------

Coordinator/researcher’s name ------------------------------------------------- Time----------

(Please print your name)

**This is only necessary when the Guardian/Parent cannot read:**

I have witnessed the information concerning this study being fully explained and understood by the Parent/Guardian and permission has been granted by the Parent/Guardian without being forced or coerced.

Witness’ signature------------------------------------Date-----------------------

Witness’ name----------------------------------------Time------------------------

(Please print your name)

- A witness is a person not involved in the study or who is not involved in obtaining consent.

The Parent/Guardian’s thumb print as named above if he /she cannot write

*Parent or Guardian is to be given a copy of the signed consent form to keep.*

APPENDIX D: **Data collection forms**

**UPLOADED SEPERATELY**
