# Supplementary material for: A reduced-carbohydrate and lactose-free formulation for stabilization among hospitalized children with severe acute malnutrition: A double-blind, randomized controlled trial
Source: PLoS Med. 2019 Feb 26;16(2):e1002747. doi: 10.1371/journal.pmed.1002747 (PMC6390989; doi:10.1371/journal.pmed.1002747)
Supplement: S1 Table — All data are median (IQR) or geometric means (95% CI), as indicated. (DOCX) [file pmed.1002747.s003.docx]

# **S1 Table Primary endpoint and pre-specified subgroup analyses**

| Days to stabilization | F75 | Modified F75 | P-Value |
| --- | --- | --- | --- |
| All participants | 3 (2-4) | 3 (2-4) | 0.59 |
| *Sub-groups** | | | |
| Diarrhoea at admission | | | |
| Absent | 3 (2-4) | 3 (2-4) | 0.58 |
| Present | 3 (2-5) | 3 (2-4) |  |
| HIV antibody test | | | |
| Negative | 3 (2-4) | 3 (2-4) | 0.13 |
| Positive | 3 (2-4.5) | 3 (2-5) |  |
| Unknown | 2.5 (2-3) | 3 (2-3) |  |
| Kwashiorkor | | | |
| Absent | 3 (2-4) | 3 (2-4) | 0.43 |
| Present | 3 (2-5) | 3 (2-5) |  |
| Age group | | | |
| <12 months | 3 (2-5) | 3 (2-4) | 0.83` |
| 12-23 months | 3 (2-4) | 3 (2-4) |  |
| ≥24 months | 3 (2-4) | 3 (2-5) |  |
| Site | | | |
| Kilifi County Hospital | 3 (2-5) | 2 (3-4) | 0.0017 |
| Coast General Hospital | 3 (2-4) | 3 (2-4) |  |
| Queen Elizabeth Central Hospital | 3 (2-4) | 3 (2-5) |  |

**Primary endpoint and pre-specified subgroup analyses**

**All data are median (interquartile range) *P-values are from likelihood ratio tests.**

|  | All participants* | Standard  F75 | Modified  F75 | P** |
| --- | --- | --- | --- | --- |
| All participants |  |  |  | 0.59 |
| Kilifi County Hospital | 3.00 (2.71-3.22) | 3.35 (2.88-3.91) | 2.69 (2.36-3.08) | 0.07 |
| Coast Provincial General Hospital | 2.79 (2.62-2.98) | 2.88 (2.61-3.17) | 2.71 (2.49-2.95) | 0.47 |
| Queen Elizabeth Central Hospital | 3.17 (2.98-3.37) | 3.00 (2.76-3.27) | 3.34 (3.05-3.66) | 0.15 |

**Primary endpoint analysed per site.**

All data are geometric means (95% CI), amongst those who survived to stabilisation

* Kruskall-Wallis test between sites P=0.03

** Wilcoxon rank sum test between allocated groups
