# Supplementary material for: A reduced-carbohydrate and lactose-free formulation for stabilization among hospitalized children with severe acute malnutrition: A double-blind, randomized controlled trial
Source: PLoS Med. 2019 Feb 26;16(2):e1002747. doi: 10.1371/journal.pmed.1002747 (PMC6390989; doi:10.1371/journal.pmed.1002747)
Supplement: S2 Table — All data are presented as median (IQR). (DOCX) [file pmed.1002747.s004.docx]

# **S2 Table Competitive Risk Models**

|  |  |  |  |  |  |  |  |  |  |
| --- | --- | --- | --- | --- | --- | --- | --- | --- | --- |
|  | Days to stabilisation | | Days to death | |  | Model 1 | | Model 2 | |
|  | F75 | mF75 | F75 | mF75 |  | HR [95% CI] | *P* | HR [95% CI] | *P* |
| Study arm | 3 [2-4] | 3 [2-4] | 4 [3-7] | 3.5 [2.3-5.8] | Study arm | 1.1 [0.73 to 1.8] | 0.58 | 0.91 [0.48 to 1.7] | 0.78 |
|  |  |  |  |  | Coastal Provincial General | 0.87 [0.52 to 1.4] | 0.57 | 0.65 [0.32 to 1.3] | 0.24 |
|  |  |  |  |  | Kilifi County | 0.65 [0.35 to 1.2] | 0.17 | 0.58 [0.24 to 1.4] | 0.23 |
|  |  |  |  |  | mF75 : Coastal Provincial General | - | - | 1.7 [0.63 to 4.7] | 0.29 |
|  |  |  |  |  | mF75 : Kilifi County | - | - | 1.2 [0.35 to 4.1] | 0.76 |
|  |  |  |  |  |  |  |  |  |  |
| Diarrhoea at admission | |  |  |  |  |  |  |  |  |
| Absent | 3 [2-4] | 3 [2-4] | 4 [3.8-7.3] | 4 [2-5.5] | Study arm | 1.2 [0.74 to 1.8] | 0.54 | 0.92 [0.46 to 1.8] | 0.81 |
| Present | 3 [2-5] | 3 [2-4] | 4 [2-5.5] | 3 [3-5.5] | Diarrhoea present | 1.4 [0.92 to 2.3] | 0.11 | 1.2 [0.63 to 2.3] | 0.57 |
|  |  |  |  |  | mF75 : diarrhoea present | - | - | 1.5 [0.60 to 3.6] | 0.4 |
|  |  |  |  |  | Coastal Provincial General | 0.80 [ 0.48 to 1.3] | 0.40 | 0.79 [ 0.47 to 1.3] | 0.37 |
|  |  |  |  |  | Kilifi County | 0.66 [0.35 to 1.2] | 0.19 | 0.66 [0.35 to 1.2] | 0.18 |
|  |  |  |  |  |  |  |  |  |  |
| HIV reactivity | |  |  |  |  |  |  |  |  |
| Negative | 3 [2-4] | 3 [2-4] | 5 [4-7] | 4 [2-7] | Study arm | 0.95 [0.60 to 1.5] | 0.81 | 1.2 [0.61 to 2.3] | 0.62 |
| Positive | 3 [2-4.3] | 3 [2-5] | 4 [2-4.5] | 4.5 [3-6.8] | HIV positive | 2.2 [1.3 to 3.7] | 0.003 | 2.7 [1.4 to 5.4] | 0.004 |
| Unknown | 2.5 [2-3] | 3 [2-3] | 3.5 [2.8-4] | 3 [2-3] | HIV unknown | 8.8 [4.1 to 19] | <0.0001 | 10 [3.7 to 27] | <0.0001 |
|  |  |  |  |  | mF75 : HIV positive | - | - | 0.62 [0.23 to 1.7] | 0.33 |
|  |  |  |  |  | mF75 : HIV unknown | - | - | 0.77 [0.20 to 3.0] | 0.70 |
|  |  |  |  |  | Coastal Provincial General | 0.69 [ 0.39 to 1.2] | 0.20 | 0.70 [ 0.40 to 1.3] | 0.23 |
|  |  |  |  |  | Kilifi County | 0.71 [0.38 to 1.3] | 0.29 | 0.70 [0.38 to 1.3] | 0.26 |
|  |  |  |  |  |  |  |  |  |  |
| Oedema |  |  |  |  |  |  |  |  |  |
| Absent | 3 [2-4] | 3 [2-4] | 4 [2.5-5] | 4 [3-6.5] | Study arm | 1.1 [0.72 to 1.8] | 0.59 | 1.3 [0.75 to 2.2] | 0.37 |
| Present | 3 [2-5] | 3 [2-5] | 4 [3-7] | 3 [2-5] | Present oedema | 0.75 [0.46 to 1.2] | 0.27 | 0.92 [0.47 to 1.8] | 0.8 |
|  |  |  |  |  | mF75 : oedema | - | - | 0.68 [0.26 to 1.8] | 0.42 |
|  |  |  |  |  | Coastal Provincial General | 0.77 [ 0.46 to 1.3] | 0.34 | 0.77 [ 0.46 to 1.3] | 0.33 |
|  |  |  |  |  | Kilifi County | 0.60 [0.32 to 1.1] | 0.11 | 0.61 [0.33 to 1.1] | 0.11 |
| Age group |  |  |  |  |  |  |  |  |  |
| <12 months | 3 [2-5] | 3 [2-4] | 5 [4 6.5] | 4 [3-5] | Study arm | 1.1 [0.73 to 1.8] | 0.58 | 1.0 [0.46 to 2.2] | 0.99 |
| 12-23 months | 3 [2-4] | 3 [2-4] | 3 [2-7] | 3 [3-5] | Age | 1.0 [0.98 to 1.0] | 0.62 | 0.99 [0.97 to 1.0] | 0.51 |
| ≥ 24 months | 3 [2-4] | 3 [2-4.25] | 4 [2.5-5.5] | 4 [2-7.5] | mF75 : Age | - | - | 1.0 [0.98 to 1.0] | 0.70 |
|  |  |  |  |  | Coastal Provincial General | 0.84 [ 0.49 to 1.4] | 0.50 | 0.83 [ 0.49 to 1.4] | 0.50 |
|  |  |  |  |  | Kilifi County | 0.64 [0.34 to 1.2] | 0.16 | 0.64 [0.34 to 1.2] | 0.16 |

All data are presented as median [IQR, interquartile range]. Hazard ratios (HR) calculated by competitive risk regression analysis for time-to-event (i.e., days to stabilization or days to death before stabilization) adjusted for site. Model 2 included interaction terms (indicated with colons) between study arms and patient subgroups as defined by diarrhea at admission, HIV reactivity, oedema status, age. Reference groups for the HR were: standard F75, Queen Elizabeth Central Hospital, diarrhea absent, HIV non-reactive, and edema absent. Significance threshold, Gray’s *P* <0.05. HR, hazard ratio; 95% CI, 95% confidence intervals; F75, standard F75; mF75, modified F75.
