# Supplementary material for: A reduced-carbohydrate and lactose-free formulation for stabilization among hospitalized children with severe acute malnutrition: A double-blind, randomized controlled trial
Source: PLoS Med. 2019 Feb 26;16(2):e1002747. doi: 10.1371/journal.pmed.1002747 (PMC6390989; doi:10.1371/journal.pmed.1002747)
Supplement: S3 Table — (DOCX) [file pmed.1002747.s005.docx]

# **Table S3 – Serious adverse events**

|  | **Site** | **Sex** | **Age months** | **HIV test** | **Attribution** | **Outcome** | **Shock** | **Sepsis** | **Hypoxia** | **Pneumonia** | **Diarrhoea** | **Anaemia** | **Cardiac  Failure** | **Impaired  Consciousness** | **Convulsions** | **Other Diagnosis** |
| --- | --- | --- | --- | --- | --- | --- | --- | --- | --- | --- | --- | --- | --- | --- | --- | --- |
|  | **ARM - Standard F75** | | | | | | | | | | | | | | | |
| 1 | QECH | Female | 26 | Negative | Not related | Resolved | No | No | No | No | Yes | No | No | No | No |  |
| 2 | QECH | Female | 7 | Negative | Indeterminate | Fatal | No | No | No | No | Yes | No | No | No | No | SEVERE METABOLIC ACIDOSIS |
| 3 | QECH | Female | 15 | Unknown | Not related | Fatal | Yes | Yes | No | No | No | No | No | No | No |  |
| 4 | QECH | Female | 29 | Negative | Indeterminate | Fatal | No | No | No | No | Yes | No | No | Yes | No | ASPIRATION PNEUMONIA |
| 5 | QECH | Female | 16 | Negative | Indeterminate | Fatal | Yes | Yes | No | No | No | Yes | No | No | No | METABOLIC ACIDOSIS |
| 6 | QECH | Female | 26 | Negative | Indeterminate | Fatal | No | No | No | No | No | Yes | No | No | No |  |
| 7 | QECH | Female | 19 | Negative | Not related | Fatal | No | No | No | No | No | No | No | No | No | ASPIRATION PNEUMONIA |
| 8 | QECH | Female | 17 | Negative | Not related | Fatal | Yes | No | No | No | No | No | No | Yes | No |  |
| 9 | QECH | Female | 10 | Negative | Indeterminate | Resolved | No | No | No | No | Yes | No | No | No | No |  |
| 10 | QECH | Female | 12 | Negative | Not related | Fatal | No | No | No | No | No | No | No | No | No | ASPIRATION PNEUMONIA |
| 11 | QECH | Female | 19 | Negative | Not related | Fatal | Yes | No | No | No | No | No | No | No | No | SEPTIC SHOCK |
| 12 | QECH | Female | 15 | Positive | Not related | Fatal | No | No | No | No | No | Yes | No | No | No |  |
| 13 | QECH | Female | 23 | Positive | Not related | Fatal | No | Yes | No | No | No | No | No | Yes | Yes | MENINGITIS |
| 14 | QECH | Female | 11 | Negative | Not related | Fatal | No | Yes | No | No | No | No | No | Yes | No | ASPIRATION PEUMONIA |
| 15 | QECH | Female | 8 | Negative | Indeterminate | Resolved | No | No | No | No | Yes | No | No | No | No |  |
| 16 | QECH | Female | 32 | Negative | Not related | Fatal | No | Yes | No | No | Yes | No | No | No | No |  |
| 17 | QECH | Female | 16 | Negative | Indeterminate | Resolved | No | No | No | No | Yes | No | No | No | No |  |
| 18 | QECH | Female | 27 | Positive | Not related | Fatal | Yes | No | No | No | No | No | No | No | No | ASPIRATION PNEUMONIA |
| 19 | QECH | Female | 55 | Negative | Not related | Resolved | No | Yes | No | Yes | No | No | No | No | No |  |
| 20 | QECH | Female | 32 | Negative | Indeterminate | Fatal | No | No | No | No | Yes | No | No | No | No |  |
| 21 | QECH | Female | 21 | Negative | Not related | Resolved | No | Yes | No | No | Yes | No | No | No | No |  |
| 22 | QECH | Female | 40 | Positive | Not related | Resolved | No | No | No | Yes | No | No | No | No | No |  |
| 23 | QECH | Female | 85 | Negative | Not related | Resolved | No | No | No | No | Yes | No | No | No | No |  |
| 24 | QECH | Female | 9 | Positive | Not related | Fatal | No | No | No | Yes | No | No | No | No | No |  |
| 25 | QECH | Female | 21 | Negative | Not related | Unresolved on discharge | No | No | No | No | No | No | No | No | No | GANGRENOUS ARM:COMPARTMENT SYNDROME |
| 26 | QECH | Female | 34 | Negative | Indeterminate | Resolved | No | No | No | No | Yes | No | No | No | No |  |
| 27 | QECH | Female | 40 | Negative | Not related | Fatal | No | No | No | No | No | No | No | No | No | ASPIRATION PNEUMONIA |
| 28 | QECH | Female | 7 | Negative | Indeterminate | Fatal | No | No | No | No | Yes | No | No | No | No | METABOLIC ACIDOSIS |
| 29 | QECH | Male | 16 | Negative | Not related | Fatal | No | No | No | No | Yes | No | No | No | No | ASPIRATION |
| 30 | QECH | Male | 16 | Negative | Not related | Fatal | No | Yes | No | No | No | No | No | No | No |  |
| 31 | QECH | Male | 17 | Negative | Indeterminate | Resolved | No | No | No | No | No | Yes | No | No | No |  |
| 32 | QECH | Male | 25 | Positive | Not related | Fatal | No | No | No | No | Yes | No | No | No | No |  |
| 33 | QECH | Male | 28 | Negative | Not related | Resolved with sequelae | No | No | No | Yes | No | No | No | No | No | TUBERCULOSIS |
| 34 | QECH | Male | 24 | Negative | Indeterminate | Resolved | No | No | No | No | Yes | No | No | No | No |  |
| 35 | QECH | Male | 18 | Positive | Indeterminate | Fatal | No | Yes | No | No | No | No | No | Yes | No | METABOLIC ACIDOSIS |
| 36 | QECH | Male | 78 | Negative | Not related | Unresolved on discharge | No | No | No | Yes | No | No | No | Yes | No | METABOLIC ACIDOSIS |
| 37 | QECH | Male | 21 | Negative | Not related | Fatal | No | No | No | No | No | Yes | No | No | No | ASPIRATION |
| 38 | QECH | Male | 33 | Negative | Not related | Resolved | No | No | No | No | No | Yes | No | No | No |  |
| 39 | QECH | Male | 8 | Positive | Not related | Fatal | No | No | No | Yes | Yes | No | No | No | No | ASPIRATION PNEUMONIA |
| 40 | QECH | Male | 45 | Positive | Indeterminate | Fatal | Yes | Yes | No | No | No | No | No | No | No | ADVANCED HIV DISEASE |
| 41 | QECH | Male | 38 | Negative | Indeterminate | Resolved | No | No | No | No | Yes | No | No | No | Yes |  |
| 42 | QECH | Male | 26 | Negative | Indeterminate | Resolved | No | Yes | No | No | No | Yes | No | No | No |  |
| 43 | QECH | Male | 21 | Positive | Not related | Resolved with sequelae | No | No | No | Yes | No | No | No | No | No |  |
| 44 | QECH | Male | 12 | Positive | Not related | Fatal | No | Yes | No | No | Yes | No | No | No | No |  |
| 45 | QECH | Male | 27 | Negative | Not related | Fatal | No | No | No | No | No | No | No | No | No | ASPIRATION PNEUMONIA |
| 46 | QECH | Male | 12 | Negative | Indeterminate | Resolved | No | No | No | No | Yes | No | No | No | No |  |
| 47 | QECH | Male | 14 | Negative | Indeterminate | Resolved | No | No | No | No | Yes | No | No | No | No |  |
| 48 | QECH | Male | 18 | Negative | Not related | Fatal | Yes | No | No | No | Yes | No | No | No | No |  |
| 49 | QECH | Male | 22 | Positive | Not related | Fatal | Yes | Yes | No | No | No | No | No | No | No |  |
| 50 | QECH | Male | 22 | Positive | Not related | Fatal | No | No | No | No | No | No | No | No | No | ADVANCED HIV DISEASE |
| 51 | QECH | Male | 11 | Positive | Indeterminate | Fatal | No | Yes | No | No | Yes | No | No | No | Yes | MENINGITIS |
| 52 | QECH | Male | 8 | Unknown | Not related | Fatal | No | No | No | Yes | No | No | No | No | No | ASPIRATION |
| 53 | QECH | Male | 41 | Unknown | Not related | Fatal | No | Yes | No | No | No | No | No | No | No | SEVERE MALARIA |
| 54 | QECH | Male | 7 | Positive | Indeterminate | Resolved | No | No | No | No | Yes | No | No | No | No |  |
| 55 | QECH | Male | 43 | Positive | Not related | Resolved | No | No | No | No | Yes | No | No | No | No |  |
| 56 | QECH | Male | 41 | Negative | Not related | Fatal | No | No | No | No | No | No | No | No | No | ASPIRATION PNEUMONIA |
| 57 | QECH | Male | 6 | Negative | Not related | Resolved | No | No | No | Yes | No | No | No | No | No |  |
| 58 | CPGH | Female | 18 | Unknown | Not related | Fatal | No | No | No | No | Yes | No | No | No | No |  |
| 59 | CPGH | Female | 101 | Positive | Not related | Fatal | No | Yes | No | Yes | No | No | No | No | No | ADVANCED HIV DISEASE |
| 60 | CPGH | Female | 9 | Unknown | Not related | Resolved | No | No | No | No | No | No | Yes | No | No | PERICARDIAL EFFUSION |
| 61 | CPGH | Female | 22 | Positive | Not related | Resolved | Yes | No | No | No | Yes | No | No | No | No | ASPIRATION PNEUMONIA |
| 62 | CPGH | Female | 9 | Positive | Not related | Fatal | No | No | No | No | No | Yes | No | No | No |  |
| 63 | CPGH | Female | 9 | Negative | Not related | Resolved | No | No | Yes | Yes | Yes | No | No | No | No |  |
| 64 | CPGH | Female | 15 | Negative | Not related | Fatal | No | Yes | No | No | No | No | No | No | No |  |
| 65 | CPGH | Female | 10 | Negative | Not related | Fatal | No | Yes | No | No | No | No | No | Yes | Yes | RENAL FAILURE |
| 66 | CPGH | Female | 11 | Unknown | Not related | Fatal | No | No | No | No | No | No | No | Yes | Yes | SEVERE MALARIA |
| 67 | CPGH | Female | 11 | Negative | Not related | Unresolved on discharge | No | No | No | No | No | No | No | No | No | TUBERCULOSIS |
| 68 | CPGH | Female | 8 | Negative | Not related | Resolved | No | No | No | Yes | No | No | No | No | No | URINARY TRACT INFECTION |
| 69 | CPGH | Female | 7 | Unknown | Not related | Fatal | No | Yes | No | No | No | No | No | No | No |  |
| 70 | CPGH | Female | 14 | Negative | Not related | Fatal | Yes | No | No | No | Yes | No | No | No | No |  |
| 71 | CPGH | Female | 8 | Negative | Not related | Fatal | No | No | No | No | No | No | No | No | No | UNKNOWN |
| 72 | CPGH | Female | 14 | Negative | Not related | Unresolved on discharge | No | No | No | No | No | No | No | No | No | TUBERCULOSIS |
| 73 | CPGH | Female | 12 | Unknown | Not related | Fatal | No | Yes | No | Yes | No | No | No | No | No |  |
| 74 | CPGH | Female | 12 | Negative | Not related | Resolved | Yes | Yes | No | No | No | No | No | No | Yes |  |
| 75 | CPGH | Female | 12 | Unknown | Not related | Fatal | No | Yes | No | No | No | No | No | No | No | MENINGITIS |
| 76 | CPGH | Female | 11 | Negative | Not related | Resolved | No | No | No | No | Yes | No | No | No | No |  |
| 77 | CPGH | Female | 10 | Unknown | Not related | Fatal | No | No | No | Yes | Yes | No | No | No | No |  |
| 78 | CPGH | Female | 11 | Negative | Not related | Fatal | No | No | No | No | No | No | No | No | No |  |
| 79 | CPGH | Female | 43 | Negative | Not related | Fatal | No | No | No | No | No | Yes | No | No | No | PANCYTOPENIA |
| 80 | CPGH | Female | 22 | Positive | Not related | Unresolved on discharge | No | No | No | No | No | No | No | No | No | TUBERCULOSIS |
| 81 | CPGH | Female | 8 | Positive | Not related | Fatal | No | No | No | Yes | No | No | No | No | No |  |
| 82 | CPGH | Male | 6 | Unknown | Not related | Fatal | No | No | No | Yes | No | No | No | No | No |  |
| 83 | CPGH | Male | 11 | Negative | Not related | Unresolved on discharge | No | No | No | No | Yes | No | No | No | No | TUBERCULOSIS |
| 84 | CPGH | Male | 10 | Negative | Not related | Fatal | No | Yes | No | No | Yes | No | No | No | No |  |
| 85 | CPGH | Male | 10 | Unknown | Not related | Resolved | No | No | No | No | No | No | Yes | No | No |  |
| 86 | CPGH | Male | 10 | Positive | Not related | Fatal | No | No | No | No | Yes | No | No | No | No |  |
| 87 | CPGH | Male | 11 | Negative | Not related | Unresolved on discharge | No | No | No | No | No | No | No | No | No | TUBERCULOSIS |
| 88 | CPGH | Male | 15 | Negative | Not related | Resolved | No | No | No | No | No | No | No | No | No |  |
| 89 | CPGH | Male | 16 | Negative | Not related | Unresolved on discharge | No | No | No | No | No | No | No | No | No | POSTERIOR URETHRAL VALVES |
| 90 | CPGH | Male | 14 | Negative | Not related | Fatal | No | Yes | No | No | No | No | No | No | Yes |  |
| 91 | CPGH | Male | 15 | Positive | Indeterminate | Resolved | Yes | No | No | No | Yes | No | No | No | Yes |  |
| 92 | CPGH | Male | 19 | Negative | Not related | Fatal | No | Yes | No | Yes | No | No | No | No | No |  |
| 93 | CPGH | Male | 6 | Positive | Not related | Fatal | Yes | Yes | No | No | No | No | No | No | No | INTUSUCCEPTION |
| 94 | KCH | Female | 8 | Positive | Not related | Fatal | No | No | No | Yes | No | No | No | Yes | Yes |  |
| 95 | KCH | Female | 12 | Negative | Not related | Fatal | No | Yes | No | Yes | No | No | No | Yes | No |  |
| 96 | KCH | Female | 15 | Negative | Not related | Resolved | Yes | No | No | No | Yes | No | No | No | No |  |
| 97 | KCH | Female | 31 | Negative | Not related | Fatal | No | No | No | Yes | No | No | No | No | No |  |
| 98 | KCH | Female | 9 | Positive | Not related | Fatal | No | No | No | No | No | No | No | No | No | ASPIRATION PNEUMONIA |
| 99 | KCH | Female | 25 | Negative | Not related | Resolved | No | Yes | No | No | No | No | No | No | No | BACTERAEMIA |
| 100 | KCH | Female | 17 | Positive | Not related | Unresolved on discharge | No | No | No | No | No | No | No | No | No | TUBERCULOSIS |
| 101 | KCH | Male | 53 | Negative | Not related | Unresolved on discharge | No | No | No | No | No | No | No | No | No | HAEMATOLOGICAL MALIGNANCY |
| 102 | KCH | Male | 42 | Negative | Not related | Fatal | No | No | No | No | No | No | No | No | No | INTESTINAL OBSTRUCTION |
| 103 | KCH | Male | 55 | Negative | Not related | Fatal | No | No | No | No | No | No | No | No | No |  |
| 104 | KCH | Male | 6 | Negative | Not related | Fatal | No | Yes | Yes | Yes | No | No | No | Yes | No |  |
| 105 | KCH | Male | 13 | Positive | Not related | Unresolved on discharge | No | No | No | No | No | No | No | No | No | TUBERCULOSIS |
| 106 | KCH | Male | 16 | Negative | Not related | Fatal | No | Yes | No | No | Yes | No | No | No | No |  |
| 107 | KCH | Male | 48 | Negative | Not related | Fatal | No | No | No | No | No | Yes | No | No | No | HYPERKALAEMIA |
| 108 | KCH | Male | 39 | Positive | Not related | Fatal | Yes | No | No | No | Yes | No | No | No | No |  |
|  | **ARM - modified F75** | | | | | | | | | | | | | | | |
| 1 | QECH | Female | 26 | Positive | Not related | Fatal | No | No | No | No | No | Yes | No | No | No | PRESUMED ASPIRATION |
| 2 | QECH | Female | 42 | Positive | Not related | Fatal | No | Yes | No | No | No | No | No | No | No | METABOLIC ACIDOSIS |
| 3 | QECH | Female | 10 | Negative | Not related | Resolved | No | No | No | No | No | No | No | No | No | SEVERE METABOLIC ACIBORIS |
| 4 | QECH | Female | 10 | Negative | Indeterminate | Resolved | No | No | No | No | Yes | No | No | No | No |  |
| 5 | QECH | Female | 10 | Negative | Indeterminate | Fatal | No | No | No | No | Yes | No | No | No | No |  |
| 6 | QECH | Female | 19 | Negative | Indeterminate | Fatal | No | Yes | No | No | Yes | No | No | No | No |  |
| 7 | QECH | Female | 12 | Positive | Indeterminate | Fatal | Yes | No | No | No | Yes | No | No | No | No |  |
| 8 | QECH | Female | 23 | Positive | Related | Resolved | No | No | No | No | Yes | No | No | No | No |  |
| 9 | QECH | Female | 19 | Negative | Not related | Fatal | No | Yes | No | No | No | No | No | No | No |  |
| 10 | QECH | Female | 14 | Negative | Indeterminate | Fatal | No | No | No | No | Yes | No | No | No | No |  |
| 11 | QECH | Female | 10 | Positive | Indeterminate | Fatal | Yes | No | No | No | Yes | No | No | No | No | METABOLIC ACIDOSIS |
| 12 | QECH | Female | 53 | Negative | Not related | Resolved | No | No | No | No | No | Yes | No | No | No |  |
| 13 | QECH | Female | 8 | Positive | Not related | Fatal | No | No | No | No | No | No | No | No | No | ASPIRATION PNEUMONIA |
| 14 | QECH | Female | 27 | Negative | Not related | Resolved | No | No | No | No | No | Yes | No | No | No |  |
| 15 | QECH | Female | 21 | Positive | Not related | Resolved | No | No | No | No | No | No | Yes | No | No |  |
| 16 | QECH | Female | 20 | Positive | Not related | Fatal | No | Yes | No | No | Yes | No | No | No | No | METABOLIC ACIDOSIS |
| 17 | QECH | Female | 18 | Negative | Not related | Resolved | No | No | No | Yes | No | No | No | No | No |  |
| 18 | QECH | Female | 12 | Negative | Not related | Resolved | No | No | No | No | Yes | No | No | No | No |  |
| 19 | QECH | Female | 15 | Positive | Not related | Fatal | No | Yes | No | No | No | No | No | No | No | METABOLIC ALKALOSIS |
| 20 | QECH | Female | 11 | Negative | Not related | Fatal | No | No | No | Yes | No | No | No | No | No | ASPIRATION PNEUMONIA |
| 21 | QECH | Female | 16 | Positive | Not related | Fatal | No | Yes | No | Yes | No | No | No | Yes | No | TUBERCULOSIS |
| 22 | QECH | Female | 22 | Negative | Indeterminate | Fatal | Yes | Yes | No | No | No | No | No | No | No | METABOLIC ACIDOSIS |
| 23 | QECH | Male | 21 | Negative | Indeterminate | Fatal | No | No | No | No | No | No | No | No | No | ASPIRATION PNEUMONIA |
| 24 | QECH | Male | 13 | Negative | Not related | Resolved | No | No | No | No | Yes | No | No | No | No |  |
| 25 | QECH | Male | 11 | Positive | Not related | Fatal | No | Yes | No | No | No | No | No | No | Yes |  |
| 26 | QECH | Male | 32 | Positive | Indeterminate | Fatal | Yes | No | No | No | No | No | No | No | No | HIV ENTEROPATHY |
| 27 | QECH | Male | 31 | Positive | Indeterminate | Fatal | No | No | No | No | Yes | No | No | No | No | HYPERNATRAEMIA |
| 28 | QECH | Male | 25 | Negative | Not related | Fatal | No | No | No | No | No | Yes | No | No | No |  |
| 29 | QECH | Male | 72 | Negative | Not related | Fatal | No | No | No | No | No | No | No | No | No | ASPIRATION PNEUMONIA |
| 30 | QECH | Male | 27 | Negative | Not related | Fatal | No | Yes | No | No | No | No | No | No | No | PERSISTENT FEVERS |
| 31 | QECH | Male | 54 | Positive | Not related | Fatal | No | Yes | No | No | No | No | No | No | No | ADVANCED HIV DISEASE |
| 32 | QECH | Male | 19 | Negative | Not related | Fatal | Yes | Yes | No | No | No | No | No | No | No | SEPTIC SHOCK |
| 33 | QECH | Male | 22 | Positive | Indeterminate | Fatal | No | Yes | No | No | No | No | No | No | No | ACIDOSIS, METABOLIC |
| 34 | QECH | Male | 30 | Negative | Indeterminate | Fatal | Yes | No | No | No | Yes | No | No | No | No |  |
| 35 | QECH | Male | 24 | Positive | Not related | Resolved | No | No | No | No | Yes | No | No | No | No |  |
| 36 | QECH | Male | 45 | Positive | Not related | Resolved | No | No | No | No | Yes | No | No | No | No |  |
| 37 | QECH | Male | 25 | Positive | Not related | Fatal | No | No | No | Yes | No | No | No | No | No |  |
| 38 | QECH | Male | 11 | Positive | Not related | Resolved | No | No | No | Yes | No | No | No | No | No | PCP PNEUMONIA |
| 39 | QECH | Male | 8 | Unknown | Not related | Fatal | No | Yes | No | Yes | No | No | No | No | No |  |
| 40 | QECH | Male | 11 | Positive | Indeterminate | Resolved | No | No | No | No | Yes | No | No | No | No |  |
| 41 | QECH | Male | 8 | Negative | Not related | Fatal | No | No | No | No | No | No | No | No | No | ASPIRATION PNEUMONIA |
| 42 | QECH | Male | 91 | Positive | Not related | Resolved | No | No | No | No | Yes | No | No | No | No |  |
| 43 | QECH | Male | 6 | Negative | Indeterminate | Fatal | No | No | No | No | Yes | No | No | No | No | METABOLIC ACIDOSIS |
| 44 | CPGH | Female | 7 | Negative | Not related | Fatal | No | No | Yes | Yes | No | No | Yes | No | No | CONGENITAL HEART DISEASE |
| 45 | CPGH | Female | 25 | Negative | Not related | Resolved | No | No | No | No | No | No | No | No | No | URINARY TRACT INFECTION |
| 46 | CPGH | Female | 86 | Unknown | Not related | Fatal | Yes | No | No | No | No | No | No | No | No | TUBERCULOSIS |
| 47 | CPGH | Female | 10 | Negative | Not related | Resolved | Yes | Yes | No | No | No | No | No | No | No |  |
| 48 | CPGH | Female | 13 | Positive | Not related | Resolved | No | No | No | Yes | No | No | No | No | No |  |
| 49 | CPGH | Female | 6 | Unknown | Not related | Fatal | No | Yes | No | No | No | No | No | No | No |  |
| 50 | CPGH | Female | 7 | Negative | Not related | Fatal | No | Yes | No | No | No | No | No | Yes | No |  |
| 51 | CPGH | Female | 12 | Negative | Not related | Unresolved on discharge | No | No | No | No | No | No | No | No | No | PERACARDIAL EFFUSION |
| 52 | CPGH | Female | 14 | Positive | Not related | Fatal | No | No | No | No | No | Yes | No | No | No | PCP PNEUMONIA |
| 53 | CPGH | Female | 8 | Negative | Not related | Unresolved on discharge | No | No | No | No | No | No | No | No | No | PERICARDIAL EFFUSION |
| 54 | CPGH | Female | 6 | Negative | Not related | Fatal | No | No | No | Yes | No | No | No | No | No | CARDIAC FAILURE |
| 55 | CPGH | Male | 7 | Negative | Not related | Resolved | Yes | No | No | No | Yes | No | No | No | No |  |
| 56 | CPGH | Male | 7 | Negative | Not related | Resolved | Yes | No | No | No | Yes | No | No | No | No |  |
| 57 | CPGH | Male | 8 | Negative | Not related | Fatal | No | Yes | Yes | Yes | No | No | No | Yes | No |  |
| 58 | CPGH | Male | 6 | Negative | Not related | Resolved | No | No | No | No | Yes | No | No | No | No | TUBERCULOSIS |
| 59 | CPGH | Male | 8 | Negative | Not related | Fatal | Yes | Yes | Yes | Yes | No | No | No | No | No |  |
| 60 | CPGH | Male | 7 | Positive | Not related | Resolved | No | No | No | Yes | No | No | No | No | No | PCP PNEUMONIA |
| 61 | CPGH | Male | 10 | Negative | Not related | Unresolved on discharge | No | No | No | No | No | No | No | No | No | TUBERCULOSIS |
| 62 | CPGH | Male | 6 | Negative | Not related | Resolved | Yes | No | No | Yes | No | No | No | No | No | DILATED, CARDIOMYOPATHY |
| 63 | CPGH | Male | 9 | Negative | Not related | Unresolved on discharge | No | No | No | No | No | No | No | No | No | TUBERCULOSIS |
| 64 | CPGH | Male | 10 | Negative | Not related | Resolved | No | No | No | No | No | Yes | No | No | No | INCREASING OEDEMA |
| 65 | CPGH | Male | 25 | Negative | Not related | Resolved | No | Yes | No | No | No | No | No | No | No |  |
| 66 | CPGH | Male | 16 | Negative | Not related | Unresolved on discharge | No | No | No | No | No | No | No | No | No | TUBERCULOSIS |
| 67 | CPGH | Male | 14 | Positive | Not related | Fatal | No | Yes | No | No | No | No | No | No | No |  |
| 68 | CPGH | Male | 11 | Negative | Not related | Fatal | No | No | No | Yes | Yes | No | No | No | No |  |
| 69 | CPGH | Male | 11 | Negative | Not related | Resolved | No | Yes | No | No | Yes | No | No | No | No |  |
| 70 | CPGH | Male | 14 | Unknown | Not related | Fatal | Yes | Yes | No | No | No | No | No | Yes | No |  |
| 71 | CPGH | Male | 12 | Negative | Not related | Fatal | Yes | No | No | No | No | Yes | No | Yes | No | LIVER FAILURE |
| 72 | CPGH | Male | 8 | Negative | Not related | Unresolved on discharge | No | No | No | No | No | No | No | No | No |  |
| 73 | CPGH | Male | 9 | Negative | Not related | Fatal | Yes | No | No | No | No | No | No | No | No |  |
| 74 | CPGH | Male | 53 | Negative | Not related | Resolved | No | Yes | No | No | No | No | No | No | No |  |
| 75 | CPGH | Male | 7 | Positive | Not related | Fatal | Yes | Yes | No | No | No | No | No | No | No |  |
| 76 | CPGH | Male | 7 | Unknown | Not related | Fatal | Yes | Yes | No | No | No | No | No | No | No |  |
| 77 | CPGH | Male | 7 | Unknown | Not related | Fatal | No | No | No | Yes | No | No | No | No | No |  |
| 78 | KCH | Female | 12 | Negative | Not related | Resolved | No | No | No | No | Yes | No | No | No | No | TUBERCULOSIS |
| 79 | KCH | Female | 43 | Negative | Not related | Fatal | No | No | No | No | No | No | No | No | No | INTESTINAL OBSTRUCTION |
| 80 | KCH | Female | 78 | Positive | Not related | Unresolved on discharge | No | No | No | Yes | No | No | No | No | No | TUBERCULOSIS |
| 81 | KCH | Female | 6 | Negative | Not related | Unresolved on discharge | No | No | No | Yes | No | No | No | No | No | TUBERCULOSIS |
| 82 | KCH | Female | 38 | Positive | Not related | Resolved | No | No | No | No | No | No | No | No | No |  |
| 83 | KCH | Female | 107 | Positive | Not related | Unresolved on discharge | No | No | No | No | No | No | No | No | No | TUBERCLOSIS |
| 84 | KCH | Female | 65 | Positive | Not related | Fatal | No | Yes | No | Yes | No | No | No | No | No | ADVANCED HIV DISEASE |
| 85 | KCH | Female | 13 | Positive | Not related | Fatal | Yes | No | Yes | Yes | Yes | No | No | No | No |  |
| 86 | KCH | Female | 31 | Positive | Not related | Fatal | Yes | No | No | No | Yes | No | No | Yes | No |  |
| 87 | KCH | Female | 14 | Positive | Not related | Fatal | Yes | No | No | Yes | Yes | No | No | Yes | No |  |
| 88 | KCH | Male | 30 | Positive | Not related | Fatal | No | No | No | No | No | No | No | No | Yes |  |
| 89 | KCH | Male | 48 | Negative | Not related | Fatal | No | No | No | No | No | No | No | Yes | No | TUBERCULOUS MENINGITIS |
| 90 | KCH | Male | 8 | Negative | Not related | Fatal | No | No | Yes | Yes | No | No | No | Yes | No |  |
| 91 | KCH | Male | 9 | Positive | Not related | Fatal | No | Yes | No | Yes | No | No | No | No | No |  |
| 92 | KCH | Male | 31 | Positive | Indeterminate | Fatal | No | No | No | No | No | No | No | No | No |  |
| 93 | KCH | Male | 13 | Negative | Not related | Fatal | Yes | No | No | No | No | No | No | No | No |  |
| 94 | KCH | Male | 24 | Negative | Not related | Fatal | Yes | No | No | No | Yes | No | No | No | No |  |
| 95 | KCH | Male | 14 | Negative | Not related | Fatal | No | Yes | No | No | No | No | No | Yes | No |  |
| 96 | KCH | Male | 7 | Negative | Not related | Unresolved on discharge | No | No | No | No | No | No | No | No | No | TUBERCULOSIS |
