# Supplementary figures and images for: A reduced-carbohydrate and lactose-free formulation for stabilization among hospitalized children with severe acute malnutrition: A double-blind, randomized controlled trial
Source: PLoS Med. 2019 Feb 26;16(2):e1002747. doi: 10.1371/journal.pmed.1002747 (PMC6390989; doi:10.1371/journal.pmed.1002747)

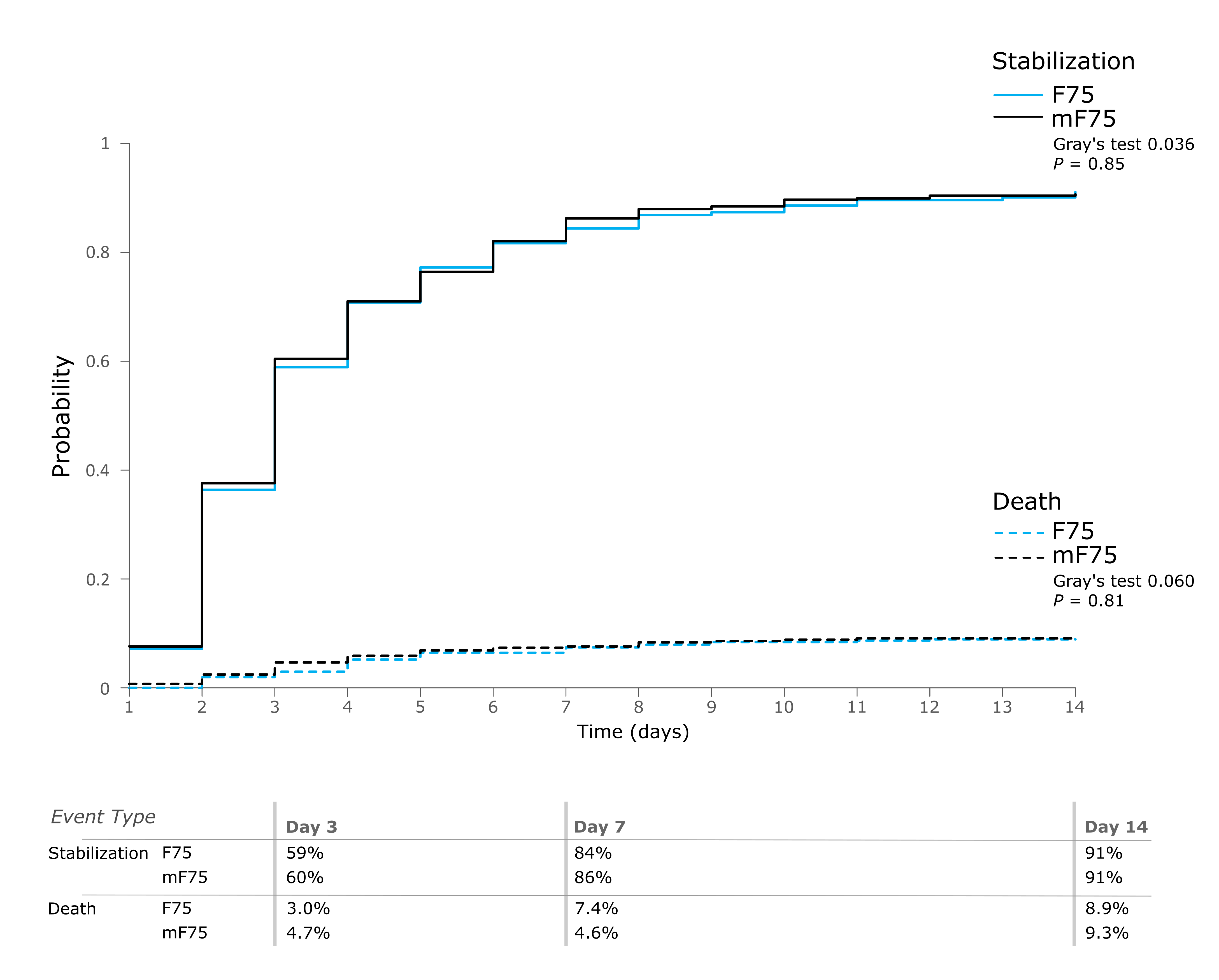

Supplement: S1 Fig — Competing risk analysis simultaneously compares the estimated cumulative incidence curves of mutually exclusive events: first stabilization (solid line) or death prior to stabilization (dashed lines). Withdrawals and absconded cases were censored. Differences in cumulative incidence functions between F75 (blue line) and mF75 (black line) and all subgroup analysis models were compared using Gray’s test. Significance threshold, P < 0.05. F75, standard F75; mF75, modified F75. (TIF) [file pmed.1002747.s006.tif]
